# Efficacy and Safety of Rivaroxaban Compared with Other Therapies Used in Patients with Peripheral Artery Disease Undergoing Peripheral Revascularization: A Systematic Literature Review and Network Meta-Analysis

**DOI:** 10.1155/2021/8561350

**Published:** 2021-08-24

**Authors:** Rupert Bauersachs, Olivia Wu, Neil Hawkins, Kevin Bowrin, Piotr Wojciechowski, Emilie Clay, Maria Huelsebeck

**Affiliations:** ^1^Klinikum Darmstadt GmbH, Darmstadt, Germany; ^2^Health Economics and Health Technology Assessment, Institute of Health and Wellbeing, University of Glasgow, Glasgow, UK; ^3^Bayer Plc, Reading, UK; ^4^Creativ-Ceutical, Krakow, Poland; ^5^Creativ-Ceutical, Paris, France; ^6^Bayer AG, Berlin, Germany

## Abstract

**Background:**

The guidelines on antithrombotic treatment in patients with symptomatic peripheral artery disease (PAD) undergoing peripheral revascularization of the lower extremities were developed based on heterogeneous trials, assessing various dose regimens and recruiting patients who were subjected to different revascularization procedures.

**Objective:**

To compare efficacy and safety of treatments used in patients with PAD undergoing peripheral revascularization accounting for between-trial heterogeneity and large dispersion of the quality of evidence.

**Methods:**

A systematic literature review of randomised controlled trials (RCTs) recruiting adult patients with PAD receiving antithrombotics was conducted until January 2020. Hazard ratios (HR) were pooled using Bayesian network meta-analysis. The estimated between-treatment effects were presented as HR together with 95% credible intervals. The base case analysis included studies recruiting patients following recent peripheral revascularization, who received treatment regimens administered within the recommended therapeutic window, while a sensitivity scenario included all identified trials.

**Results:**

Thirteen RCTs were identified (8 RCTs enrolled patients following peripheral revascularization and 5 RCTs regardless of the previous revascularization). Five trials, recruiting an overall of 8349 patients, were considered for the base case analysis. Of those, 6564 patients were recruited in the VOYAGER PAD trial comparing rivaroxaban plus aspirin (RIV plus ASA) versus ASA. RIV plus ASA was associated with a lower risk of repeated peripheral revascularization versus ASA monotherapy (HR = 0.88 [0.79, 0.99]), however having a trend towards an increased rate of major bleeding (HR = 1.43 [0.98, 2.11]). There was no evidence for differences between RIV plus ASA and dual antiplatelet therapy and vitamin K antagonists plus ASA. Similar results were observed in sensitivity analyses.

**Conclusions:**

RIV plus ASA is associated with reduced risk of revascularization compared with ASA monotherapy, but the evidence for other comparators, in particular antiplatelet regimens, was insufficient to guide treatment decisions and highlights the challenge in establishing the magnitude of comparative efficacy using existing RCTs.

## 1. Introduction

Peripheral artery disease (PAD) is a common cardiovascular (CV) system disease with the global prevalence exceeding 200 million people and incidence of about 11 million in 2017 [1, 2]. Progressive atherosclerosis causes stenosis or occlusion of peripheral arteries. Depending on the location and severity of the stenosis, the disease is asymptomatic or symptomatic with intermittent claudication (IC) or atypical leg pain, critical limb ischemia (CLI), and acute limb ischemia (ALI) as typical signs [3–6]. CLI leads to chronic hypoperfusion resulting in a substantial increase in the risk of limb loss [7–9]. For this reason, patients with CLI or the occurrence of lifestyle-limiting symptoms classified as being grade IIb according to the Fontaine classification may require revascularization of the lower extremity arteries [10, 11]. Revascularization immediately improves quality of life in PAD patients and improves long-term survival [12], although it is associated with a postprocedural increase of vascular risk [13].

The clinical trials on the antithrombotic medication differ noticeably in terms of quality and sample size, with limited evidence for the efficacy and safety of antiplatelet medication used in patients who have undergone revascularization, in particular for those after recent endovascular procedures [4]. Due to this reason, the recommendations of practice guidelines were often developed based on low-quality evidence [4]. A single antiplatelet therapy (SAPT) with acetylsalicylic acid (ASA) or clopidogrel (CLO) alone is recommended for all patients with symptomatic PAD who have undergone revascularization to reduce the risk of atherothrombotic events (Class I with level of evidence C). Dual antiplatelet therapy (DAPT), consisting of low doses of ASA in combination with a P2Y12 receptor antagonist, is recommended for at least one month for patients following endovascular procedures (Class IIa with level of evidence C). DAPT with ASA plus CLO for at least 1 month can be considered in patients undergoing bypass surgery with a prosthetic graft (Class IIb with level of evidence B) [4]. Despite existing guidelines, a comparison of treatments for PAD patients after peripheral revascularization encounters difficulties mainly due to small population of PAD patients enrolled in the studies and cooccurrence of PAD and coronary artery disease. Additionally, different revascularization procedures, including surgical and endovascular, were used in respective trials. Of note, the available endovascular procedures evolved over the years which additionally increase heterogeneity. Rivaroxaban (RIV), a selective direct inhibitor of factor Xa, plus ASA is the most recent treatment option studied for PAD patients. Based on the COMPASS trial in patients suffering from chronic coronary artery disease and/or PAD, RIV plus ASA was included in the guidelines as an antithrombotic therapy in lower extremity artery disease [4]. RIV plus ASA was also recently assessed in another large clinical trial (VOYAGER PAD) in patients with symptomatic PAD undergoing surgical or endovascular revascularization [14]. In both studies, RIV plus ASA was compared to ASA alone; there are no data comparing efficacy and safety of RIV plus ASA with other therapies (e.g., clopidogrel and VKA). Therefore, the objective of this analysis was to review available evidence for treatments used in patients with PAD undergoing peripheral revascularization procedures of the lower extremities and conduct a network meta-analysis (NMA) to estimate relative efficacy and safety.

## 2. Material and Methods

### 2.1. Systematic Literature Review

A systematic literature review (SLR) was conducted to identify randomised controlled trials (RCTs) on adult patients with acute symptomatic PAD, presenting the clinical evidence for the following therapeutic options: RIV plus ASA, CLO plus ASA, ASA monotherapy, CLO monotherapy, vitamin K antagonists (VKAs) with or without ASA or CLO, and a placebo. The search was conducted in January 2020 in MEDLINE, MEDLINE In-Process, EMBASE, and CENTRAL. No timeframe and geographical scope restrictions were imposed; however, for conferences/congresses searches, only records issued from 2015 on were considered. Inclusion criteria are summarised in Table 1.

The selection of RCTs was conducted by 2 analysts working independently, and any disagreements between the 2 analysts were resolved by a third analyst. Data from studies meeting all inclusion criteria were extracted by one analyst; its quality was thoroughly checked by the second analyst. The risk of bias was assessed for each study using the tool developed by the Cochrane Collaboration [15].

### 2.2. Additional Criteria for Inclusion in the Network Meta-Analysis

The intention of this analysis was to compare the RIV plus ASA regimen with treatments recommended by the current clinical practice guidelines to treat patients with PAD after revascularization. We considered all RCTs assessing antiplatelet therapies or anticoagulant treatment enrolling patients with PAD of lower extremity with and without a history of recent revascularization to increase the power of this NMA [4–6]. Based on clinical practice guidelines, the following therapies were considered as comparators for RIV plus ASA: ASA monotherapy, CLO monotherapy, CLO in combination with ASA, VKA monotherapy, and VKA plus ASA [4].

Treatments were compared regarding clinically relevant outcomes reported across analysed studies [14]. Efficacy outcomes included IS, any stroke, MI, CV death, coronary heart death, all-cause death, major amputations (between-trial heterogeneity of definition), revascularization, venous thromboembolism (VTE), and hospitalization. Major bleeding (MB) was considered as a safety outcome; however, the definitions of this event varied across studies (Supplementary Table 7). Therefore, the comparison regarding major bleeding needs to be interpreted with caution.

### 2.3. Data Synthesis

Two trials assessing RIV plus ASA in patients with PAD were identified through the SLR [14, 16]. Both compared a combination of RIV plus ASA versus ASA alone. There are no studies comparing efficacy and safety of RIV-based treatment to other therapies mentioned in the clinical guidelines. In 11 RCTs (BOA [17], CAPRIE [18], CASPAR [19], Cassar 2005 [20], CHARISMA [21], COMPASS [16], Eikelboom 2005 [22], Johnson 2002&2004 [23], MIRROR [24], VOYAGER PAD [14], and WAVE [25]), patients allocated to reference arms received ASA monotherapy, and in 9 of those 11 trials, ASA was also administered as a combination therapy in the second arm. ASA monotherapy serves as a common reference treatment in the majority of studies, thus forming a network of evidence and allowing an indirect-treatment comparison using an NMA.

An NMA in Bayesian framework was used to compare RIV plus ASA versus other treatments [26]. The NMA is an extension of traditional pairwise meta-analysis which uses Markov chain Monte Carlo sampling to compare different treatments assessed in different studies, if they form a connected network of evidence.

The model inputs are natural logarithms of hazard ratio (log(HR)) for between-treatment comparison and the corresponding SE(log(HR)). When respective trials reported estimates calculated after several interim analyses, only final estimates corresponding to the longest available follow-up were included for the NMA. Neither of the included trials reported information suggesting violation of proportionality of hazards. For studies not reporting HRs, they were estimated by fitting the exponential survival curve to the reported number of events.

Three chains were initiated with noninformative priors and run in parallel for each analysis. The convergence between chains was checked using the Brooks-Gelman-Rubin diagnostic and trace plots. There were no closed loops in the evidence networks; thus, there was no reason for the analysis of consistency.

The NMA was conducted using fixed- and random-effect models with deviance information criterion (DIC) as the indicator of model fit to clinical data. The fixed-effect model was preferred; however, the random-effect model was considered relevant when associated with DIC value lower by ≥5 points [27]. The results are presented as HR together with corresponding 95% credible intervals (95% CI). Ranking probabilities of all treatments used the surface under the cumulative ranking area (SUCRA).

## 3. Results

### 3.1. SLR Results

**Figure 1:**
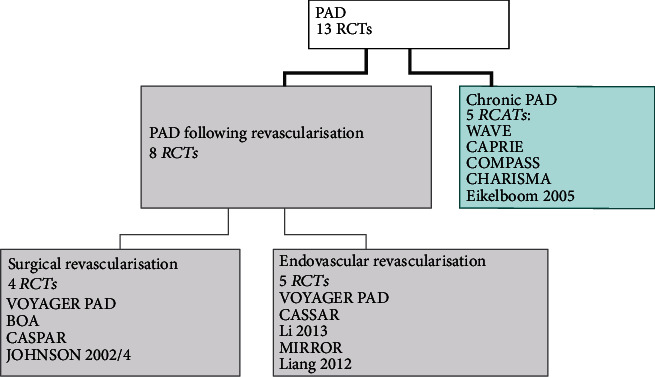
Distribution of clinical trials according to the history and type of revascularization.

Overall, 14 RCTs were identified, including a small, phase II study (RIVAL PAD [28]), which did not report any events relevant for this analysis and thus was finally excluded from the NMA. Therefore, a total of 13 RCTs were considered relevant for inclusion in the NMA (Supplementary Table 1). Of those, 8 RCTs (VOYAGER PAD [14], BOA [17], CASPAR [19], Johnson 2002&2004 [23], Cassar 2005 [20], Li 2013 [29], MIRROR [24], and Liang 2012 [30]) recruited patients following peripheral revascularization procedures of the lower extremities. Three studies (BOA [17], CASPAR [19], and Johnson 2002&2004 [23]) assessed patients after recent surgical procedures, four (Cassar 2005 [20], Li 2013 [29], MIRROR [24], and Liang 2012 [30]) recruited participants who have undergone endovascular procedures, and one (VOYAGER PAD) recruited a large sample representative for patients who have undergone either surgical or endovascular procedures. The remaining 5 studies (CAPRIE [18], COMPASS [16], CHARISMA [21], Eikelboom 2005 [22], and WAVE [25]) enrolled patients with a diagnosis of PAD regardless of previous revascularization (Table 2).  Figure 1 shows a distribution of clinical trials according to the history and type of revascularization.

Input data for the NMA is presented in Supplementary Tables 1–8. Identified studies differed in terms of methodological quality, of population characteristics, of procedure types, of follow-up duration, of ASA doses, of history of previous revascularization procedure, and of concomitant CAD and differed regarding major bleeding definition (Supplementary Table 7) and additional treatments contributing to between-trial heterogeneity (Table 2). Moreover, in the light of the rapid development of technologies for endovascular revascularization, all the identified studies assessing P2Y12 receptor antagonist in this group of patients, which were published between 2005 and 2013, may not be representative for the contemporary procedures.

#### 3.1.1. Risk of Bias

The risk of bias was evaluated for all included trials and summarised in Supplementary Table 1. There was no evidence for the risk of bias arising from the randomisation, although Li 2013 did not disclose all necessary details of the randomisation process. All trials comparing different antiplatelet regimens were conducted in a double-blinded fashion, while studies assessing anticoagulation were open label, which is justified given the need for close monitoring of INR in patients receiving VKA. Baseline characteristics were generally well balanced between the study groups; in some studies, there were differences however with no statistically significant interaction with the reported results. There was a higher proportion of patients with a history of coronary artery disease and/or cerebrovascular disease in the CLO treatment arm in the CASPAR trial and a higher proportion of patients with coronary artery disease in the antiplatelet arm in the WAVE trial.

#### 3.1.2. Population

Eleven out of 13 clinical trials enrolled just patients with lower extremity PAD; however, the protocols of 2 RCTs (COMPASS [16]; WAVE [25]) allowed also the inclusion of patients with carotid artery disease.

As mentioned before, the included trials differed regarding a history of the previous revascularization, type of procedures, etc. Eight out of the 13 RCTs were designed to assess therapeutic effects following recent peripheral revascularization. They recruited patients who recently underwent revascularization using either surgical (4 RCTs: VOYAGER PAD [14], BOA, CASPAR [19], and Johnson 2002&2004 [23]) or endovascular procedures (5 RCTs: VOYAGER PAD [14], CASSAR, Li 2013 [29], MIRROR [24], and Liang 2012 [30]) or hybrid procedure (VOYAGER). Studies just including PAD patients after a recent endovascular revascularization were small, with the number of recruited patients ranging from 56 to 132. The VOYAGER PAD trial [14] assessed RIV plus ASA in patients after surgical and/or endovascular procedures. The randomisation in this trial was stratified by procedure type and CLO use into surgical revascularization and endovascular procedures with or without concomitant CLO; hybrid procedure was considered as endovascular procedure.

Six trials (VOYAGER PAD [14], BOA [17], CASPAR [19], Cassar 2005 [20], MIRROR [24], and Li 2013 [29]) provided the number of days, within which the treatment was initiated after the revascularization. In general, the first dose of the investigated treatment was administered within 10 days after the procedure. In one RCT (Johnson 2002&2004 [23]), the treatment with warfarin was administered as soon as patients tolerated oral fluids. The remaining trial (Li 2012) stated that the treatment was initiated following angioplasty.

The 5 remaining trials (WAVE [25], CAPRIE [18], COMPASS [16], CHARISMA [21], and Eikelboom 2005 [22]) recruited patients with chronic PAD. These trials allowed to include patients with or without a history of peripheral revascularization. Prior procedure was not required for enrolment, and the time since revascularization was quite inconsistent. Three (COMPASS [16], CHARISMA [16], and Eikelboom 2005 [22]) out of these 5 RCTs recruiting patients with chronic PAD reported history of peripheral revascularization with a range of 27% to 58% (Table 2).

#### 3.1.3. Interventions and Comparators

Out of the 5 RCTs (BOA [17], Johnson 2002&2004 [23], Li 2013 [29], and Liang 2012 [30]) assessing VKA-based regimens in 2 RCTs (BOA [17]; WAVE [25]), the target INR was 2.0-3.0 and 3.0 to 4.5, respectively. In 11 RCTs (BOA [17], CAPRIE [18], CASPAR [19], Cassar 2005 [20], CHARISMA [21], COMPASS [16], Eikelboom 2005 [22], Johnson 2002&2004 [23], MIRROR [24], VOYAGER PAD [14], and WAVE [25]), patients allocated to the reference arm received either ASA monotherapy and/or ASA combined with other treatments. There was between-trial heterogeneity regarding ASA doses. A high ASA daily dose (325 mg) was administered in 2 trials (CAPRIE [18], Johnson 2002&2004 [23]); a broad range of ASA daily doses (81-325 mg) was allowed in the WAVE trial [25]; and low ASA dose (≤100 mg) was administered in the remaining trials.

In the WAVE trial [25], around 7% of participants received concomitant thienopyridines in either group, which further increased the level of heterogeneity associated with the assessed treatments. There was no analysis stratified by clopidogrel use in the WAVE trial that could assess the potential impact of thienopyridine use on the efficacy and safety results. Similarly, patients assigned to endovascular procedures of the VOYAGER PAD trial were stratified according to clopidogrel use (endovascular procedure with or without clopidogrel), but finally, no significant interaction between clopidogrel use and the outcomes was observed, between-trial heterogeneity.

The analysis of heterogeneity revealed noticeable between-trial differences, which could potentially interact with the estimates of the treatment effects and bias the inference based on the results of the NMA. The most important sources of heterogeneity were rooted in population differences, in procedure differences, e.g., different ASA doses and different treatment strategies. Due to the between-trial heterogeneity, two variants of the analysis were chosen.

*(1) Base Case*. The aim of this analysis was to assess between-treatment differences in efficacy and safety based on RCTs with a high level of homogeneity regarding the target population and assessing treatment regimens administered in accordance with clinical practice guidelines [4]. Therefore, this analysis was conducted only on studies recruiting patients following recent revascularization, who received treatment regimens administered within the recommended therapeutic doses.

There was limited number of studies meeting the inclusion criteria for the base case analysis; thus, the comparison with some of the predefined comparators including VKA monotherapy, CLO monotherapy, and the combination treatment including CLO plus ASA was not feasible.

*(2) Sensitivity Analysis*. The aim of this analysis was to allow for the comparison versus comparators not assessed in the base case and analyse the robustness of the comparative results based on relaxed inclusion/exclusion criteria while acknowledging the heterogeneity introduced with that. This analysis was conducted based on the 13 studies included in the NMA.

### 3.2. Results of the NMA

#### 3.2.1. Base Case Analysis

Fixed-effect models were considered for each comparison based on DIC criterion and considering that in most cases only one study was available for each comparison; therefore, the heterogeneity parameter could not be estimated. A consistency analysis was not conducted as all the networks had star-like shape without closed loops allowing to compare between direct and indirect evidence.

The trials eligible for the base case analysis allowed to form a network of evidence linking RIV plus ASA (VOYAGER PAD) with CLO plus ASA (CASPAR, Cassar 2005, MIRROR) and VKA plus ASA (Johnson 2002&2004) through ASA monotherapy. RIV plus ASA and CLO plus ASA could formally be compared regarding all outcomes of interest except for ALI, which was assessed only in the VOYAGER PAD trial [14]. On the contrary, the Johnson et al.'s trial (Johnson 2002&2004) [23] which compared VKA plus ASA and ASA reported only all-cause death, any stroke, MI, and major bleeding; therefore, the comparison was limited to reported outcomes.

Consistent with the results of the VOYAGER PAD trial [14], this analysis showed that RIV plus ASA compared with ASA monotherapy is associated with a significantly lower risk of unplanned index-limb revascularization for recurrent ischemia. The comparison between RIV plus ASA with DAPT (CLO plus ASA) and warfarin-based regimen did not reveal any significant differences between treatments regarding safety and efficacy outcomes. However, a trend in favour of RIV plus ASA was observed for comparison with VKA plus ASA regarding all-cause deaths (HR = 0.77 [0.57, 1.04]) (Table 3).

#### 3.2.2. Sensitivity Analysis

Additional trials included in the sensitivity analysis allowed for the assessment of relative treatment effects also versus CLO monotherapy and VKA monotherapy, although the former option was only the comparison for CV deaths, IS, any stroke, and the risk of MI.

Consistent with the base case analysis, RIV plus ASA was associated with a lower risk of peripheral revascularization compared with ASA monotherapy (HR = 0.89 [0.81, 0.98]). For the majority of the remaining outcomes, there was no significant difference between RIV plus ASA and ASA except for the risk of major bleeding, which was significantly higher in the RIV plus ASA group (HR = 1.52 [1.17, 1.98]) compared to ASA alone.

The NMA did not reveal significant treatment differences, with the exception for the comparison with VKA plus ASA regarding major bleeding; there was a lower risk associated with RIV plus ASA (HR = 0.54 [0.33, 0.89]).

The inclusion of additional studies enrolling stable population leads to a nonsignificant modification of the relative effects. The results of the CHARISMA trial [21] favoured CLO plus ASA over ASA regarding all-cause death, CV death, and major bleeding; thus, the point estimates for comparison between RIV plus ASA versus CLO plus ASA favoured the latter treatment regarding these outcomes, although all estimates were not significant.

There were no other major differences in the direction and significance of the effect estimates between the base case scenario and the sensitivity analysis, which could modify the inference.

Input data on amputations for the NMAs are presented in Table 4. Input data on other outcomes for the NMAs (Supplementary Tables 1–8), figures presenting networks of evidence for all outcomes (Supplementary Figures 1, 3, 5, 7, 9, 11, 13, and 15), and forest plots (Supplementary Figures 2, 4, 6, 8, 10, 12, 14, and 16) are presented in Supplementary Materials. The results of the base case scenario and sensitivity analysis are shown in Table 3.

## 4. Discussion

With this NMA, we attempted to summarise existing evidence on efficacy and safety profiles of treatments used in patients with PAD undergoing peripheral revascularization and accounting for the between-trial heterogeneity and large dispersion of the quality of evidence.

The SLR conducted to collect data for the NMA revealed scarcity and serious limitations of available clinical data. We identified only 5 RCTs reporting adequate results that were eligible for the base case analysis and allowed to compare RIV plus ASA only with 2 regimens: CLO plus ASA and VKA plus ASA. Of those, the VOYAGER PAD study was the largest trial enrolling 6564 out of 8449 patients recruited in all studies included for the base case scenario. The study was designed and powered to compare RIV plus ASA versus ASA in a double-blinded fashion regarding clinically relevant outcomes and showed a significant reduction of the primary efficacy endpoint defined as a composite of acute limb ischemia, major amputation for vascular causes, myocardial infarction, ischemic stroke, or death from cardiovascular causes. All participants underwent a recent peripheral revascularization prior to a random allocation to treatment groups, which was stratified according to the type of revascularization procedure (endovascular (including hybrid) vs. surgical) and according to clopidogrel use among those who underwent an endovascular procedure [14]. The primary ITT analysis was based on the primary efficacy composite endpoint, and several secondary outcomes were tested in a hierarchical order. The principal safety outcome was major bleeding according to the Thrombolysis in Myocardial Infarction (TIMI) classification. The results of the VOYAGER PAD trial showed that RIV plus ASA reduced the risk of the primary efficacy outcome by 15% compared with ASA monotherapy (HR = 0.85 [0.76, 0.96]; *p* = 0.009) with no significant increase of the rate of TIMI major bleeding (HR = 1.43 [0.97, 2.10]; *p* = 0.07) and a significant increase in ISTH major bleeding events (HR = 1.42 [1.10, 1.84]; *p* = 0.007).

While the large VOYAGER study provided high-quality results for assessing the clinical effectiveness of RIV plus ASA, the evidence for other therapeutic options in a postrevascularization setting is limited. ASA and ASA plus CLO were compared in patients after endovascular procedures in only 2 RCTs (Cassar 2005 [20]; MIRROR [24]) recruiting 103 and 68 patients, respectively. Both RCTs were designed to assess platelet functions and had inadequate power for the evaluating clinically relevant outcomes. Moreover, both studies were conducted before 2012 and thus may not represent the contemporary endovascular procedures, which evolved in the recent years.

One trial (CASPAR [19]) compared CLO plus ASA versus ASA monotherapy in 851 patients following surgical revascularization. This study did not demonstrate a benefit of CLO plus ASA over ASA regarding the primary endpoint, defined as a composite of index-graft occlusion or revascularization, above-ankle amputation of the affected limb, or death (HR = 0.98 [0.78-1.23]). A post hoc analysis in the subset of patients with prosthetic grafts showed a difference in favour of the CLO plus ASA regimen (HR = 0.65 [0.45-0.95]). Finally, the remaining trial included in the base case compared VKA plus ASA versus ASA in 831 PAD patients after a surgical revascularization. The study was designed to assess graft patency and mortality. The results indicated that a combination of VKA plus ASA is associated with increased mortality (HR = 1.41 [1.09, 1.84]) and elevated risk of major bleeding (*p* = 0.02) including intracranial bleed, hospitalization for bleeding, an operation for control of bleeding, or a blood transfusion. Neither of the trials identified through an SLR provided clinical evidence for the efficacy of CLO monotherapy in patients following revascularization; thus, the evidence supporting the use in that population is questionable.

This NMA suggests that RIV in combination with ASA is associated with reduced risk of peripheral revascularization compared with ASA monotherapy, but there seems to be no differences between RIV plus ASA and other treatment options. However, such an indirect comparison has major limitation due to the high heterogeneity of the included trials. The sensitivity analysis with broader inclusion criteria, but with an even more increased heterogeneity, came to the same result. With one exception, the inclusion of additional trials led to an increased estimate of the risk of major bleeding in the VKA plus ASA group compared with RIV plus ASA. The results of this NMA were still imprecise since not all studies included in the network were of sufficient quality and power to draw reliable conclusions based on indirect treatment comparison. Included trials were not powered for the assessment of the individual clinical outcomes but were designed to assess either composite outcomes or surrogates. The results of the sensitivity analysis were associated with high uncertainty due to associated between-trial heterogeneity. Therefore, although this NMA was conducted in accordance with the highest methodological principles, the available clinical evidence is insufficient to draw reliable conclusions regarding the relative effectiveness and safety of the therapies used in patients after revascularization of peripheral arteries.

The main limitations of the identified scientific evidence are the inadequate samples of included trials and the related insufficient statistical power to assess respective clinically relevant outcomes. In addition, this NMA with a star-like evidence network inherited the limitations from the individual pairwise comparisons, which means that the quality of the comparison between RIV plus ASA and comparators is limited by the quality of evidence for comparators. Thus, small trials on comparators, not always designed to assess clinically relevant outcomes, are insufficient to draw reliable conclusions regarding relative efficacy and safety between the treatments used in clinical practice in this target population. The low power of this analysis and the between-trial heterogeneity with high probability explains the lack of significant differences between treatments assessed within this NMA. The definition of major bleeding was heterogeneous across included studies (Supplementary Table 7), although there was no evidence that the definition of major bleeding events could affect the relative difference between groups expressed with hazard ratios. Moreover, the risk of bleeding is expected to increase with the dose/number of antiplatelet therapies and the intensity of anticoagulation treatments. Since the daily dose of ASA ranged from 75 mg to 325 mg and the target INR also differed across studies assessing anticoagulation, the between-treatment differences regarding the risk of major bleeding should be interpreted with adequate caution. Despite including additional studies, the sensitivity analysis also did not allow a reliable and unequivocal conclusion regarding treatment differences and did not noticeably change the results of the primary analysis.

In summary, the results of this NMA are considered imprecise due to the low power of some of the included trials to assess relevant clinical outcomes and due to the high between-trial heterogeneity. Therefore, although this NMA was conducted in accordance with high methodological principles, no reliable conclusions regarding efficacy and safety of therapies in symptomatic PAD patients after a recent revascularization can be drawn. To assess clinical efficacy and safety of, for example, clopidogrel or other anticoagulant therapies in such a population, well-powered, randomised double-blind clinical studies like the VOYAGER trial are required.

## 5. Conclusions

There was limited clinical evidence for the therapies used in the treatment of patients with PAD undergoing peripheral revascularization procedures of the lower extremities. Of these, RIV plus ASA was assessed in the largest clinical program on over 11,500 patients with PAD, of which 6564 were enrolled following recent peripheral revascularization. This analysis suggests that RIV in combination with ASA is associated with reduced risk of revascularization compared with ASA monotherapy, but the evidence for comparators was insufficient to improve treatment decisions and highlights the challenge in establishing the magnitude of comparative treatment effects using existing RCT data. Robust evaluation of clinical outcomes may therefore require extending the scope of the evaluation beyond RCT data.

## Figures and Tables

**Table 1:** Inclusion criteria for the systematic literature review according to PICOS methodology.

PICOS	Inclusion criteria
Population	Adults (≥18 years) with acute symptomatic PAD
Intervention/comparators	RIV+ASA±CLO ASA CLO CLO+ASA VKA monotherapy VKA±(ASA or CLO)
Outcomes of interest	Efficacy including the following: ALI, major amputation, MI, IS, CV death Hospitalization for a coronary or peripheral cause (either lower limb) of a thrombotic nature All-cause death Safety including the following: Bleeding (major, minor, requiring medical attention, GI, intracranial, and fatal) AEs (severe, treatment-emergent) Haemorrhagic stroke Thrombotic thrombocytopenic purpura
Study designs	Study type: RCTs All languages

AE: adverse event; ALI: acute limb ischemia; ASA: aspirin; CLO: clopidogrel; CV: cardiovascular; IS: ischemic stroke; MI: myocardial infarction; PAD: peripheral artery disease; RCT: randomised controlled trial; VKA: vitamin K antagonists.

**Table 2:** Characteristics of eligible studies on PAD patients meeting inclusion criteria for NMA.

Trial name	Treatment	Reference	Sample	Patients after revascularization	Design	Follow-up (months)
PAD after revascularization: surgical and endovascular						
VOYAGER PAD (2020) [14]	RIV 2.5 mg bid+ASA (100 mg)	ASA (100 mg)	6564	100% (recent)^∗^	Double blind	Median: 28
PAD after revascularization: surgical						
BOA (2000) [17]	Phenprocoumon/Acenocoumarol (INR 3.0-4.5)	ASA (80 mg)	2650	100% (recent)^∗^	Open label	Mean: 21
CASPAR (2010) [19]	CLO+ASA (75-100 mg)	ASA (75-100 mg)	851		Double blind	Median: 11.9
Johnson 2002/2004 [23]	Warfarin (INR 1.4-2.8)+ASA (325 mg)	ASA (325 mg)	831		Open label	Mean: 36.6
PAD after revascularization: endovascular						
Cassar 2005 [20]	CLO+ASA (75 mg)	ASA (75 mg)	132	100% (recent)^∗^	Double blind	1
Li 2013 [29]	CLO+warfarin (INR 1.8-2.5)	CLO	88		Open label	≤12
MIRROR (2012) [24]	CLO+ASA (100 mg)	ASA (100 mg)	80		Double blind	6
Liang 2012 [30]	CLO+warfarin (INR 1.8-2.5)	CLO	56		Open label	≤12
Chronic PAD patients						
CAPRIE (1996) [18]	CLO+placebo	ASA (325 mg)	19,185 (PAD: 6452)	Not reported	Double blind	Mean: 22.9
COMPASS (2017) [16]	RIV (2.5 mg bid)+ASA (100 mg)	ASA (100 mg)	27,395 (PAD: 7470)	27.4%	Double blind	Mean: 23
	RIV (5 mg bid)					
CHARISMA (2006) [21]	CLO+ASA (75-162 mg)	ASA (75-162 mg)	15,603 (PAD: 3096)	50.6%	Double blind	Median: 28
Eikelboom (2005) [22]	CLO+ASA (100 mg)	ASA (100 mg)	36	58.3%	Double blind	3 weeks
WAVE (2007) [25]	VKA (INR 2.0-3.0)+ASA (81-325 mg)	ASA (81-325 mg)	2161	Not reported	Open label	Mean: 35

AE: adverse event; ALI: acute limb ischemia; ASA: aspirin; bid: twice a day; CLO: clopidogrel; CV: cardiovascular; INR: international normalised ratio; IS: ischemic stroke; MI: myocardial infarction; NMA: network meta-analysis; PAD: peripheral artery disease; pt-yrs: patient-years; RCT: randomised controlled trial; VKA: vitamin K antagonists; ^∗^treatment was initiated soon after revascularization, not later than 10 days following the procedure.

**Table 3:** The results of the base case scenario and sensitivity analysis.

Hazard ratios [95% CI] for comparison between RIV+ASA and respective comparators											
		Base case scenario				Sensitivity analysis					
		ASA	CLO+ASA	VKA+ASA	DIC	ASA	CLO	CLO+ASA	VKA	VKA+ASA	DIC
All-cause death	FE RE	1.08 [0.92, 1.27] 1.08 [0.00, 245.10]	0.79 [0.42, 1.50] 0.99 [0.00, 1241.02]	0.77 [0.57, 1.04] 0.77 [0.00, 1621.02]	3.788 4.491	1.02 [0.90, 1.17] 1.01 [0.54, 1.83]	No data for comparator	1.08 [0.81, 1.42] 1.02 [0.41, 2.38]	1.00 [0.80, 1.26] 0.99 [0.34, 2.78]	0.84 [0.66, 1.06] 0.83 [0.35, 1.95]	1.704 1.832
CV death	FE RE	1.14 [0.93, 1.40] 1.14 [0.00, 582.71]	0.77 [0.37, 1.60] 0.77 [0.00, 5617.07]	No data for comparator	1.117 1.140	1.04 [0.87, 1.24] 1.00 [0.17, 5.56]	1.34 [0.97, 1.84] 1.28 [0.06, 26.01]	1.04 [0.73, 1.47] 0.93 [0.07, 9.48]	1.11 [0.83, 1.48] 1.06 [0.05, 20.50]	1.00 [0.69, 1.46] 0.96 [0.05, 18.43]	1.685 1.295
Ischemic stroke	FE RE	0.87 [0.63, 1.20] 0.87 [0.00, 484.60]	0.30 [0.01, 7.80] 0.30 [0.00, 3557.10]	No data for comparator	5.049 5.038	0.77 [0.59, 1.004] 0.72 [0.06, 8.32]	0.77 [0.51, 1.17] 0.73 [0.01, 50.42]	0.91 [0.53, 1.55] 0.76 [0.01, 19.23]	1.53 [0.81, 2.90] 1.43 [0.02, 100.50]	1.20 [0.67 2.14] 1.13 [0.01, 83.53]	8.610 9.004
Any stroke	FE RE	0.94 [0.70, 1.28] 0.94 [0.00, 508.30]	0.92 [0.35, 2.43] 0.93 [0.00, 7213.05]	0.89 [0.50, 1.61] 0.89 [0.00, 6641.02]	3.540 3.543	0.80 [0.62, 1.04] 0.76 [0.30, 1.74]	0.81 [0.54, 1.21] 0.77 [0.16, 3.36]	0.97 [0.61, 1.55] 0.90 [0.23, 2.95]	1.09 [0.66 1.80] 1.03 [0.21, 4.61]	0.78 [0.51 1.19] 0.74 [0.20, 2.44]	5.093 5.814
Amputation	FE RE	0.89 [0.68, 1.16] 0.89 [0.01, 89.00]	1.25 [0.76, 2.07] 1.19 [0.00, 331.20]	No data for comparator	1.795 3.019	0.80 [0.63, 1.02] 0.75 [0.26, 1.85]	No data for comparator	1.13 [0.73, 1.76] 1.03 [0.25, 3.37]	0.89 [0.62, 1.28] 0.84 [0.15, 4.12]	1.20 [0.48, 3.05] 1.13 [0.17, 6.44]	5.918 6.952
Myocardial infarction	FE RE	0.88 [0.70, 1.12] 0.89 [0.00, 475.10]	1.09 [0.42, 2.85] 1.11 [0.00, 8665.02]	0.69 [0.40, 1.19] 0.70 [0.00, 4836.02]	2.976 2.998	0.84 [0.69, 1.02] 0.83 [0.40, 1.69]	1.34 [0.93, 1.92] 1.32 [0.37, 4.59]	1.29 [0.84, 1.96] 1.24 [0.40, 3.45]	1.22 [0.73, 2.05] 1.20 [0.33, 4.41]	0.88 [0.62, 1.25] 0.84 [0.29, 2.25]	2.441 3.948
Peripheral revascularization	FE RE	0.88 [0.79, 0.99] 0.88 [0.00, 189.60]	1.04 [0.75, 1.46] 1.44 [0.00, 1401.00]	No data for comparator	2.510 1.765	0.89 [0.81, 0.98] 0.90 [0.42, 1.95]	No data for comparator	1.13 [0.88, 1.45] 1.17 [0.51, 4.18]	0.94 [0.80, 1.10] 0.94 [0.25, 3.61]	0.99 [0.63, 1.58] 1.00 [0.25, 3.98]	-2.239 -0.605
Major bleeding	FE RE	1.43 [0.98, 2.11] 1.42 [0.00, 752.41]	0.80 [0.25 2.56] 0.79 [0.00, 5627.25]	0.61 [0.30, 1.24] 0.60 [0.00, 4198.05]	4.727 4.760	1.52 [1.17, 1.98] 1.52 [0.67, 3.45]	No data for comparator	1.40 [0.81, 2.43] 1.34 [0.36, 4.14]	0.78 [0.51, 1.17] 0.77 [0.19, 3.10]	0.54 [0.33, 0.89] 0.54 [0.16, 1.74]	4.053 5.770

ASA: aspirin; CLO: clopidogrel; DIC: deviance information criterion; FE: fixed effect; RE: random effect; VKA: vitamin K antagonists. Note: the risk of ALI was not reported in the comparator trials; therefore, the comparison regarding this outcome was not feasible.

**Table 4:** Input data for the NMA of the risk of amputation.

Study	Treatments	Event rate *n*/*N* (%)	HR [95% CI]	Analysis	
				Base case	Sensitivity
MIRROR [24]	CLO+ASA	6/35 (17.1%)	0.94 [0.30, 2.91]	✔	✔
	ASA	6/33 (18.2%)	Reference		
CASPAR [19]	CLO+ASA	31/425 (7.3%)	0.68 [0.43, 1.08]	✔	✔
	ASA	45/426 (10.6%)	Reference		
VOYAGER PAD [14]	RIV+ASA	103/3286 (3.1%)	0.89 [0.68, 1.16]	✔	✔
	ASA	115/3278 (3.5%)	Reference		
BOA [17]	VKA	100/1326 (7.5%)	0.90 [0.69, 1.19]	✗	✔
	ASA	110/1324 (8.3%)	Reference		
CHARISMA [21]	CLO+ASA	12/1545 (0.8%)	0.71 [0.34, 1.48]	❌	✔
	ASA	17/1551 (1.1%)	Reference		
COMPASS [16]	RIV+ASA	20/2492 (0.8%)	0.53 [0.31, 0.91]	❌	✔
	ASA	38/2504 (1.5%)	Reference		
WAVE [25]	VKA+ASA	8/1080 (0.7%)	0.67 [0.27, 1.63]	❌	✔
	ASA	12/1081 (1.1%)	Reference		

✔: study eligible for the analysis; ❌: study ineligible for the analysis. ASA: acetylsalicylic acid; CLO: clopidogrel; RIV: rivaroxaban; VKA: vitamin K antagonist.

## Data Availability

The input data for NMAs used to support the findings of this study are included within the supplementary information file.

## Supplementary Materials

Supplementary Materials Supplementary Table 1: credibility assessment of trials included in the NMA [1]. Supplementary Table 2: input data for the NMA of the risk of myocardial infarction. Supplementary Table 3: input data for the NMA of the risk of ischemic stroke. Supplementary Table 4: input data for the NMA of the risk of cardiovascular death. Supplementary Table 5: input data for the NMA of the risk of all-cause mortality. Supplementary Table 6: input data for the NMA of the risk of any stroke. Supplementary Table 7: input data for the NMA of the risk of major bleeding. Supplementary Table 8: input data for the NMA of the risk of revascularization. Supplementary Figure 1: networks of evidence for the risk of amputations. Supplementary Figure 2: forest plots comparing RIV plus ASA versus comparators regarding amputation. Supplementary Figure 3: networks of evidence for the risk of myocardial infarction. Supplementary Figure 4: forest plots comparing RIV plus ASA versus comparators regarding myocardial infarction. Supplementary Figure 5: networks of evidence for the risk of ischemic stroke. Supplementary Figure 6: forest plots comparing RIV plus ASA versus comparators regarding ischemic stroke. Supplementary Figure 7: networks of evidence for the risk of cardiovascular death. Supplementary Figure 8: forest plots comparing RIV plus ASA versus comparators regarding cardiovascular death. Supplementary Figure 9: networks of evidence for the risk of all-cause mortality. Supplementary Figure 10: forest plots comparing RIV plus ASA versus comparators regarding all-cause mortality. Supplementary Figure 11: networks of evidence for the risk of any stroke. Supplementary Figure 12: forest plots comparing RIV plus ASA versus comparators regarding any stroke. Supplementary Figure 13: networks of evidence for the risk of major bleeding. Supplementary Figure 14: forest plots comparing RIV plus ASA versus comparators regarding major bleeding. Supplementary Figure 15: networks of evidence for the risk of revascularization. Supplementary Figure 16: forest plots comparing RIV plus ASA versus comparators regarding revascularization.
