# Supplementary material for: Efficacy and Safety of Rivaroxaban Compared with Other Therapies Used in Patients with Peripheral Artery Disease Undergoing Peripheral Revascularization: A Systematic Literature Review and Network Meta-Analysis
Source: Cardiovasc Ther. 2021 Aug 24;2021:8561350. doi: 10.1155/2021/8561350 (PMC8407972; doi:10.1155/2021/8561350)
Supplement: Supplementary Materials — Supplementary Table 1: credibility assessment of trials included in the NMA [1]. Supplementary Table 2: input data for the NMA of the risk of myocardial infarction. Supplementary Table 3: input data for the NMA of the risk of ischemic stroke. Supplementary Table 4: input data for the NMA of the risk of cardiovascular death. Supplementary Table 5: input data for the NMA of the risk of all-cause mortality. Supplementary Table 6: input data for the NMA of the risk of any stroke. Supplementary Table 7: input data for the NMA of the risk of major bleeding. Supplementary Table 8: input data for the NMA of the risk of revascularization. Supplementary Figure 1: networks of evidence for the risk of amputations. Supplementary Figure 2: forest plots comparing RIV plus ASA versus comparators regarding amputation. Supplementary Figure 3: networks of evidence for the risk of myocardial infarction. Supplementary Figure 4: forest plots comparing RIV plus ASA versus comparators regarding myocardial infarction. Supplementary Figure 5: networks of evidence for the risk of ischemic stroke. Supplementary Figure 6: forest plots comparing RIV plus ASA versus comparators regarding ischemic stroke. Supplementary Figure 7: networks of evidence for the risk of cardiovascular death. Supplementary Figure 8: forest plots comparing RIV plus ASA versus comparators regarding cardiovascular death. Supplementary Figure 9: networks of evidence for the risk of all-cause mortality. Supplementary Figure 10: forest plots comparing RIV plus ASA versus comparators regarding all-cause mortality. Supplementary Figure 11: networks of evidence for the risk of any stroke. Supplementary Figure 12: forest plots comparing RIV plus ASA versus comparators regarding any stroke. Supplementary Figure 13: networks of evidence for the risk of major bleeding. Supplementary Figure 14: forest plots comparing RIV plus ASA versus comparators regarding major bleeding. Supplementary Figure 15: networks of evidence for the risk o [file 8561350.f1.zip › PAD_NMA-Cardiovascular Theraputics-supplement.docx]

# Supplement to the paper: Efficacy and safety of rivaroxaban compared with other therapies used in patients with peripheral artery disease undergoing peripheral revascularization – a systematic literature review and network meta-analysis

Supplementary Table 1. Credibility assessment of trials included in the NMA[1]

| Trial name (acronym) | Was randomisation carried out appropriately? | Was the concealment of treatment allocation adequate? | Were the groups similar at the outset of the study in terms of prognostic factors? | Were the care providers, participants, and outcome assessors blind to treatment allocation? | Were there any unexpected imbalances in drop-outs between groups? | Is there any evidence to suggest that the authors measured more outcomes than they reported? | Did the analysis include an intention-to-treat analysis? If so, was this appropriate and were appropriate methods used to account for missing data? |
| --- | --- | --- | --- | --- | --- | --- | --- |
| WAVE[2] | Yes | Yes | Not clear | No | No | No | Yes |
| CASPAR[3] | Yes | Yes | Not clear | Yes | No | No | Yes |
| CHARISMA[4] | Yes | Yes | Yes | Yes | Not clear | No | Yes |
| BOA[5] | Yes | Yes | Yes | No | No | No | Yes |
| VOYAGER PAD[6] | Yes | Yes | Yes | Yes | No | No | Yes |
| CAPRIE[7] | Yes | Yes | Yes | Yes | No | Not clear | Yes |
| Cassar 2005[8] | Yes | Yes | Yes | Yes | No | Not clear | Yes |
| Eikelboom, 2005[9] | Yes | Yes | Not clear | Yes | No | Not clear | No |
| COMPASS[10] | Yes | Yes | Yes | Yes | No | No | Yes |
| Johnson, 2002&2004[11] | Yes | Yes | Not clear | No | No | Not clear | Yes |
| Li, 2013[12] | Not clear | Not clear | Yes | No | No | Not clear | No |
| Liang, 2012[13] | Yes | Not clear | Yes | Not clear | No | No | No |
| MIRROR[14] | Yes | Not clear | Not clear | Yes | No | Not clear | Yes |

Supplementary Figure 1. Networks of evidence for the risk of amputations


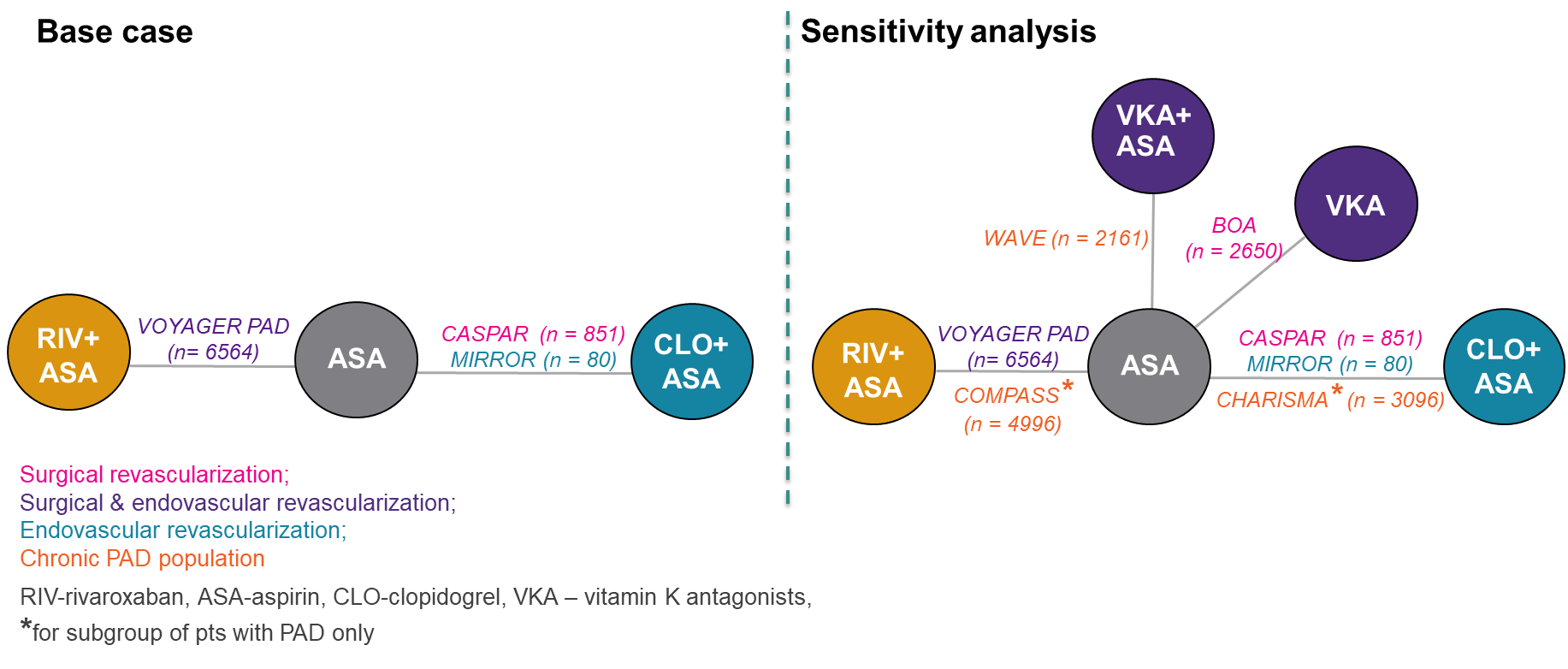


Supplementary Figure 2. Forest plots comparing RIV plus ASA versus comparators regarding amputation


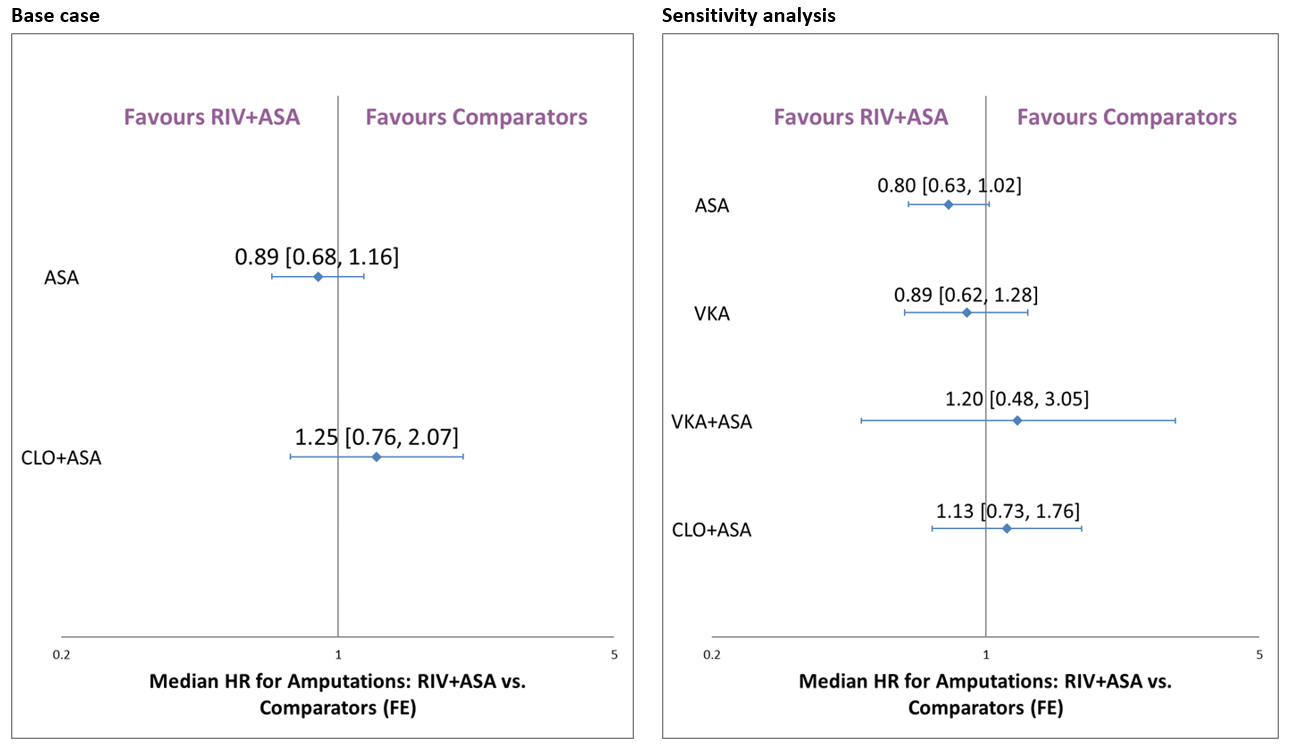


ASA, acetylsalicylic acid; FE, fixed effect; RIV, rivaroxaban.

Supplementary Table 2. Input data for the NMA of the risk of myocardial infarction

| Study | Treatments | Event rate  n/N (%) | HR [95%CI] | Analysis | |
| --- | --- | --- | --- | --- | --- |
|  |  |  |  | **Base case** | **Sensitivity** |
| CASPAR[3] | CLO+ASA | no data | 0.81 [0.32, 2.06] | ✔ | ✔ |
|  | ASA | no data | Reference |  |  |
| CAPRIE[7] | CLO | 68/3223 (2.1%) | 0.63 [0.46, 0.85] | ❌ | ✔ |
|  | ASA | 108/3229 (3.3%) | Reference |  |  |
| Johnson, 2002&2004[11] | VKA+ASA | 37/418 (8.9%) | 1.27 [0.78, 2.07] | ✔ | ✔ |
|  | ASA | 29/413 (7.0%) | Reference |  |  |
| VOYAGER PAD[6] | RIV+ASA | 131/3286 (4.0%) | 0.88 [0.70, 1.12] | ✔ | ✔ |
|  | ASA | 148/3278 (4.5%) | Reference |  |  |
| BOA[5] | VKA | 29/1326 (2.2%) | 0.69 [0.42, 1.10] | ❌ | ✔ |
|  | ASA | 42/1324 (3.2%) | Reference |  |  |
| CHARISMA[4] | CLO+ASA | 36/1545 (2.3%) | 0.63 [0.42, 0.96] | ❌ | ✔ |
|  | ASA | 57/1551 (3.7%) | Reference |  |  |
| COMPASS[10] | RIV+ASA | 51/2492 (2.0%) | 0.76 [0.53, 1.09] | ❌ | ✔ |
|  | ASA | 67/2504 (2.7%) | Reference |  |  |
| WAVE[2] | VKA+ASA | 54/1080 (5.0%) | 0.82 [0.57, 1.18] | ❌ | ✔ |
|  | ASA | 66/1081 (6.1%) | Reference |  |  |

✔– study eligible for the analysis; ❌– study ineligible for the analysis

ASA, acetylsalicylic acid; CLO, clopidogrel; RIV, rivaroxaban; VKA, vitamin K antagonist.

Supplementary Figure 3. Networks of evidence for the risk of myocardial infarction


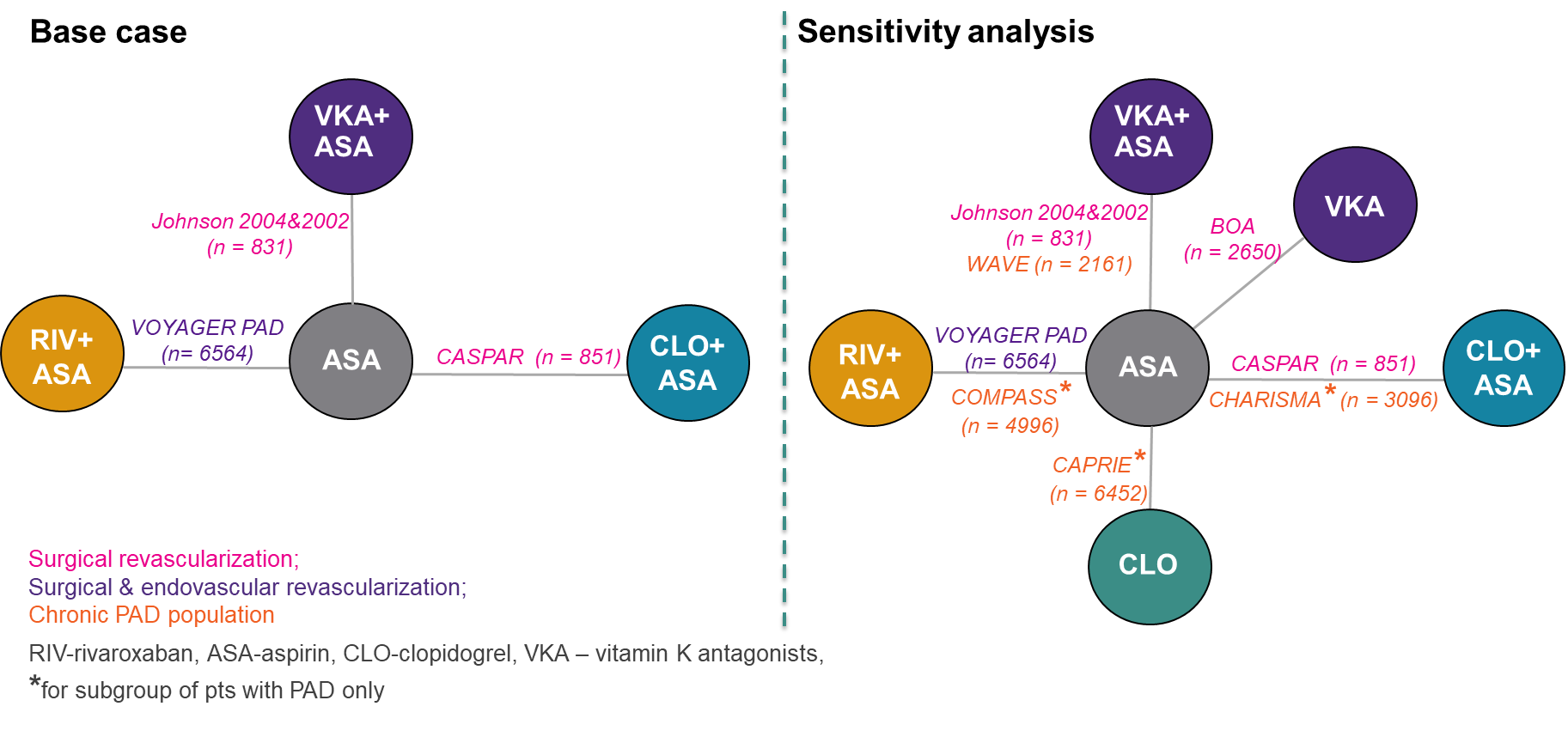


Supplementary Figure 4. Forest plots comparing RIV plus ASA versus comparators regarding myocardial infarction


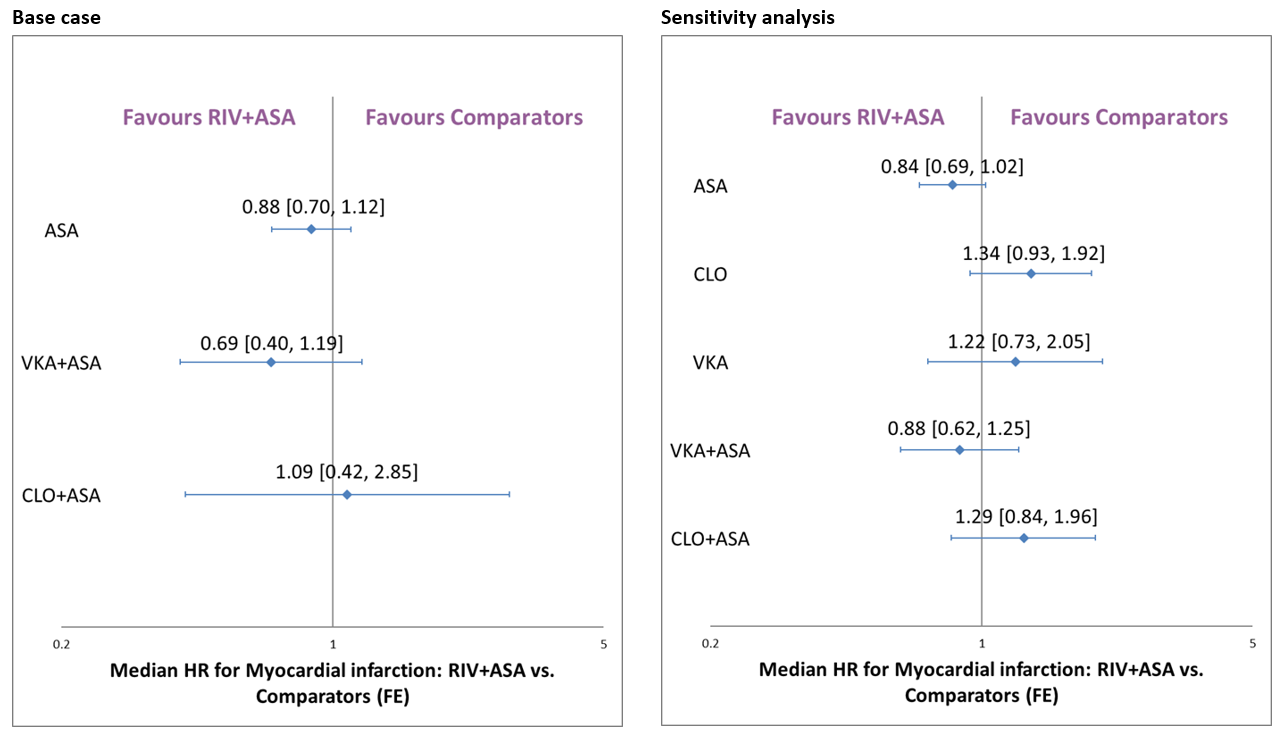


ASA, acetylsalicylic acid; FE, fixed effect; RIV, rivaroxaban.

Supplementary Table 3. Input data for the NMA of the risk of ischaemic stroke

| Study | Treatments | Event rate  n/N (%) | HR [95%CI] | Analysis | |
| --- | --- | --- | --- | --- | --- |
|  |  |  |  | **Base case** | **Sensitivity** |
| CAPRIE[7] | CLO | 81/3223 (2.5%) | 0.99 [0.73, 1.35] | ❌ | ✔ |
|  | ASA | 82/3229 (2.5%) | Reference |  |  |
| Cassar 2005[8] | CLO+ASA | 1/54 (1.9%) | 2.93 [0.11, 76.07] | ✔ | ✔ |
|  | ASA | 0/49 (0.0%) | Reference |  |  |
| VOYAGER PAD[6] | RIV+ASA | 71/3286 (2.2%) | 0.87 [0.63, 1.19] | ✔ | ✔ |
|  | ASA | 82/3278 (2.5%) | Reference |  |  |
| BOA[5] | VKA | 17/1326 (1.3%) | 0.50 [0.28, 0.89] | ❌ | ✔ |
|  | ASA | 34/1324 (2.6%) | Reference |  |  |
| CHARISMA[4] | CLO+ASA | 32/1545 (2.1%) | 0.82 [0.52, 1.32] | ❌ | ✔ |
|  | ASA | 39/1551 (2.5%) | Reference |  |  |
| COMPASS[10] | RIV+ASA | 22/2492 (0.9%) | 0.55 [0.33, 0.93] | ❌ | ✔ |
|  | ASA | 40/2504 (1.6%) | Reference |  |  |
| WAVE[2] | VKA+ASA | 24/1080 (2.2%) | 0.64 [0.38, 1.06] | ❌ | ✔ |
|  | ASA | 38/1081 (3.5%) | Reference |  |  |

✔– study eligible for the analysis; ❌– study ineligible for the analysis

ASA, acetylsalicylic acid; CLO, clopidogrel; RIV, rivaroxaban; VKA, vitamin K antagonist.

Supplementary Figure 5. Networks of evidence for the risk of ischaemic stroke


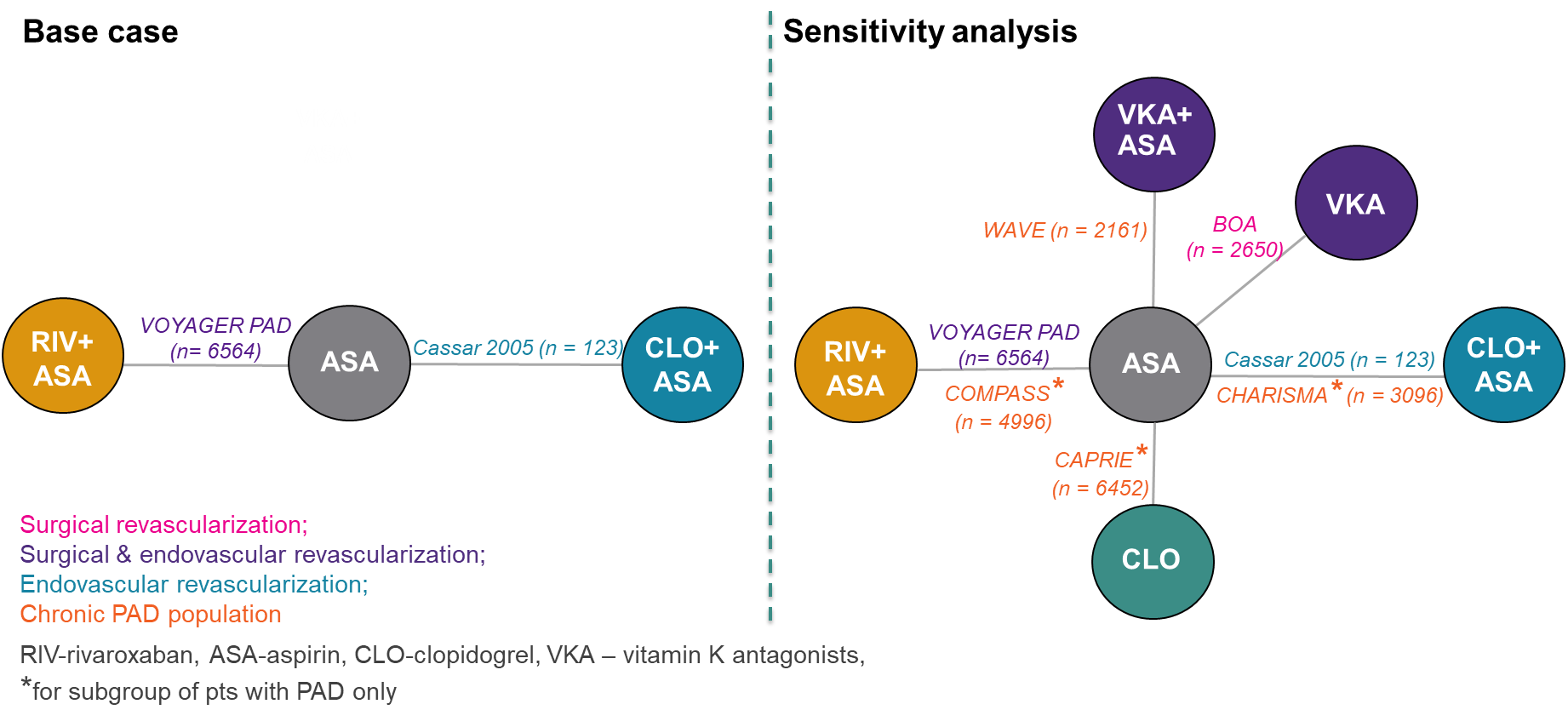


Supplementary Figure 6. Forest plots comparing RIV plus ASA versus comparators regarding ischaemic stroke


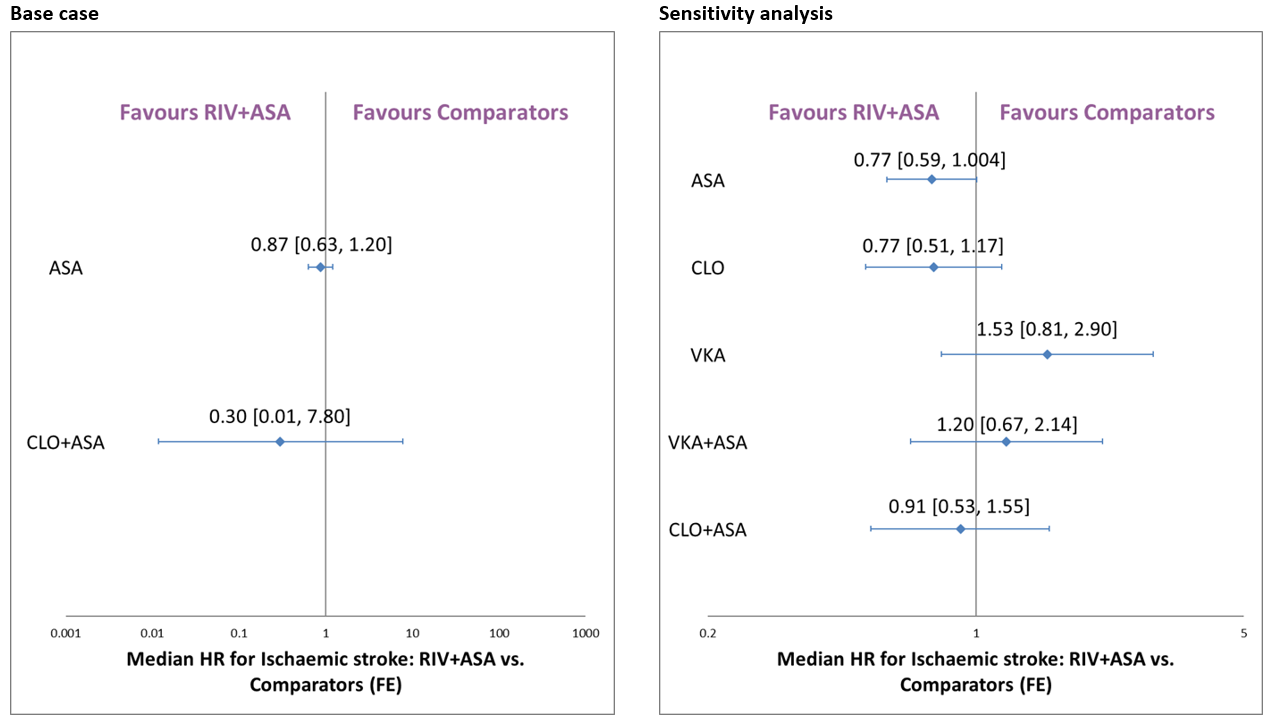


ASA, acetylsalicylic acid; FE, fixed effect; RIV, rivaroxaban.

Supplementary Table 4. Input data for the NMA of the risk of cardiovascular death

| Study | Treatments | Event rate  n/N (%) | HR [95%CI] | Analysis | |
| --- | --- | --- | --- | --- | --- |
|  |  |  |  | **Base case** | **Sensitivity** |
| CAPRIE[7] | CLO | 95/3223 (3.0%) | 0.78 [0.59, 1.02] | ❌ | ✔ |
|  | ASA | 122/3229 (3.8%) | Reference |  |  |
| CASPAR[3] | CLO+ASA | NA/425 (NA%) | 1.49 [0.73, 3.01] | ✔ | ✔ |
|  | ASA | NA/426 (NA%) | Reference |  |  |
| VOYAGER PAD[6] | RIV+ASA | 199/3286 (6.1%) | 1.14 [0.93, 1.4] | ✔ | ✔ |
|  | ASA | 174/3278 (5.3%) | Reference |  |  |
| BOA[5] | VKA | 137/1326 (10.3%) | 0.94 [0.74, 1.18] | ❌ | ✔ |
|  | ASA | 146/1324 (11.0%) | Reference |  |  |
| CHARISMA[4] | CLO+ASA | 65/1545 (4.2%) | 0.92 [0.65, 1.28] | ❌ | ✔ |
|  | ASA | 71/1551 (4.6%) | Reference |  |  |
| COMPASS[10] | RIV+ASA | 64/2492 (2.6%) | 0.82 [0.59, 1.14] | ❌ | ✔ |
|  | ASA | 78/2504 (3.1%) | Reference |  |  |
| WAVE[2] | VKA+ASA | 66/1080 (6.1%) | 1.04 [0.74, 1.46] | ❌ | ✔ |
|  | ASA | 65/1081 (6.0%) | Reference |  |  |

✔– study eligible for the analysis; ❌– study ineligible for the analysis

ASA, acetylsalicylic acid; CLO, clopidogrel; RIV, rivaroxaban; VKA, vitamin K antagonist.

Supplementary Figure 7. Networks of evidence for the risk of cardiovascular death


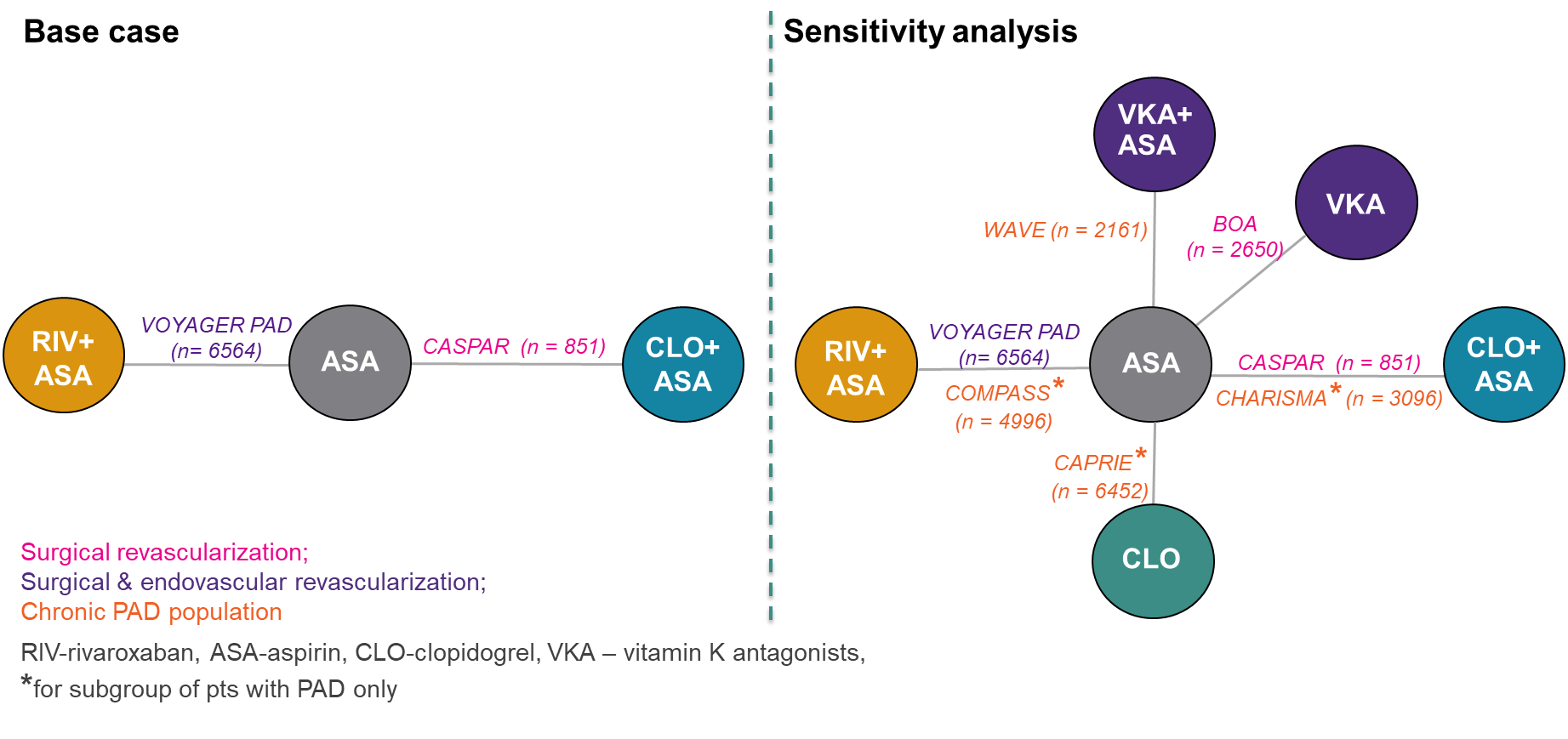


Supplementary Figure 8. Forest plots comparing RIV plus ASA versus comparators regarding cardiovascular death


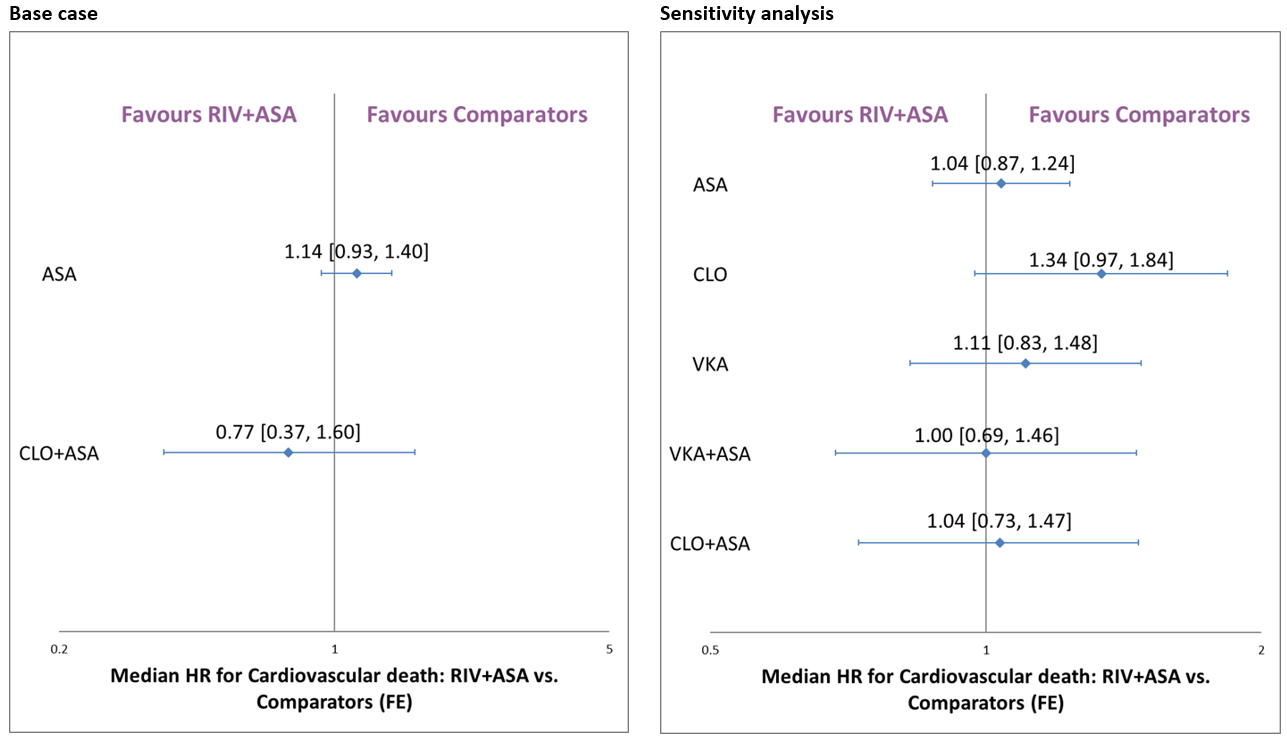


ASA, acetylsalicylic acid; FE, fixed effect; RIV, rivaroxaban.

Supplementary Table 5. Input data for the NMA of the risk of all-cause mortality

| Study | Treatments | Event rate  n/N (%) | HR [95%CI] | Analysis | |
| --- | --- | --- | --- | --- | --- |
|  |  |  |  | **Base case** | **Sensitivity** |
| MIRROR[14] | CLO+ASA | 0/40 (0.0%) | 0.33 [0.01, 8.08] | ✔ | ✔ |
|  | ASA | 1/40 (2.5%) | Reference |  |  |
| CASPAR[3] | CLO+ASA | 24/425 (5.6%) | 1.44 [0.77, 2.68] | ✔ | ✔ |
|  | ASA | 17/426 (4.0%) | Reference |  |  |
| Johnson, 2002&2004[11] | VKA+ASA | 133/418 (31.8%) | 1.41 [1.09, 1.84] | ✔ | ✔ |
|  | ASA | 95/413 (23.0%) | Reference |  |  |
| VOYAGER PAD[6] | RIV+ASA | 321/3286 (9.8%) | 1.08 [0.92, 1.27] | ✔ | ✔ |
|  | ASA | 297/3278 (9.1%) | Reference |  |  |
| BOA[5] | VKA | 211/1326 (15.9%) | 1.02 [0.85, 1.24] | ❌ | ✔ |
|  | ASA | 205/1324 (15.5%) | Reference |  |  |
| CHARISMA[4] | CLO+ASA | 104/1545 (6.7%) | 0.89 [0.68, 1.16] | ❌ | ✔ |
|  | ASA | 117/1551 (7.5%) | Reference |  |  |
| COMPASS[10] | RIV+ASA | 129/2492 (5.2%) | 0.91 [0.72, 1.16] | ❌ | ✔ |
|  | ASA | 142/2504 (5.7%) | Reference |  |  |
| WAVE[2] | VKA+ASA | 99/1080 (9.2%) | 1.04 [0.79, 1.38] | ❌ | ✔ |
|  | ASA | 96/1081 (8.9%) | Reference |  |  |

✔– study eligible for the analysis; ❌– study ineligible for the analysis

ASA, acetylsalicylic acid; CLO, clopidogrel; RIV, rivaroxaban; VKA, vitamin K antagonist.

Supplementary Figure 9. Networks of evidence for the risk of all-cause mortality


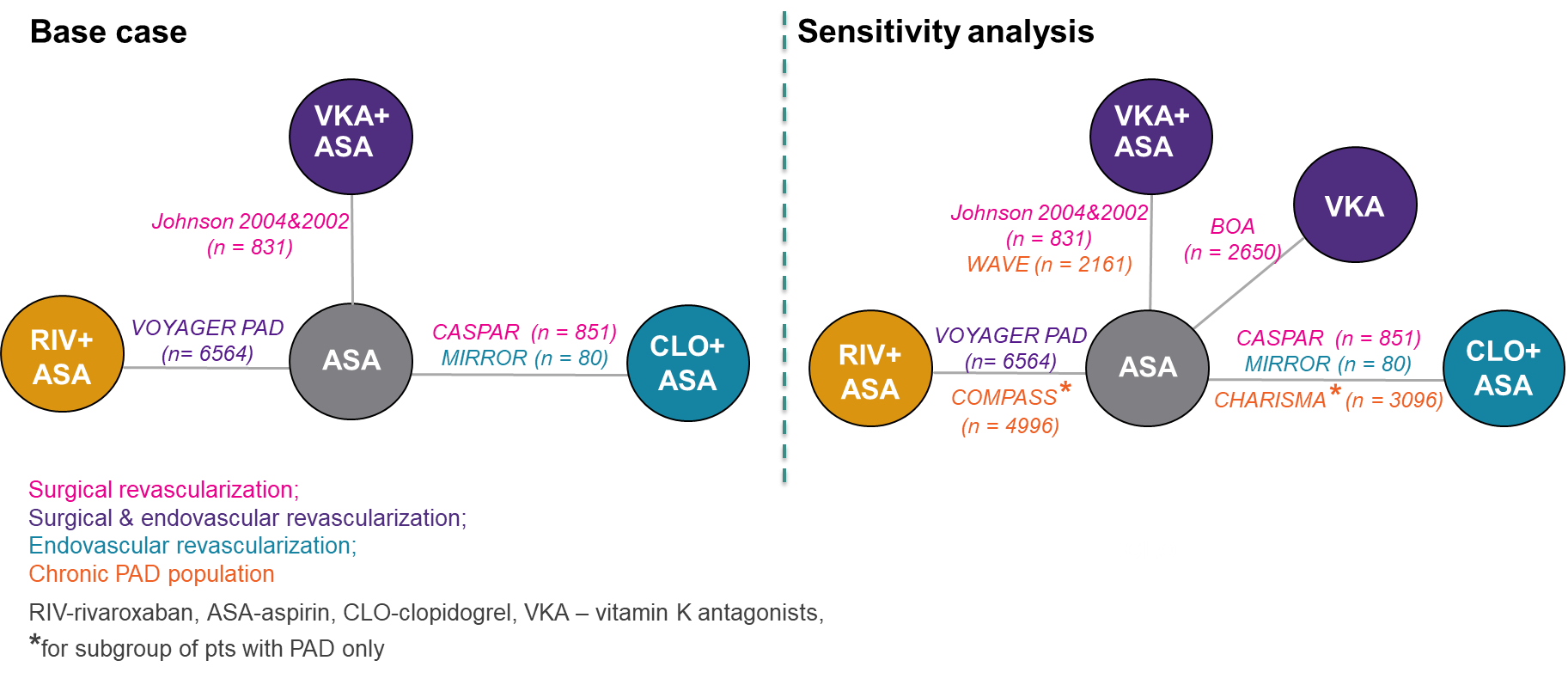


Supplementary Figure 10. Forest plots comparing RIV plus ASA versus comparators regarding all-cause mortality


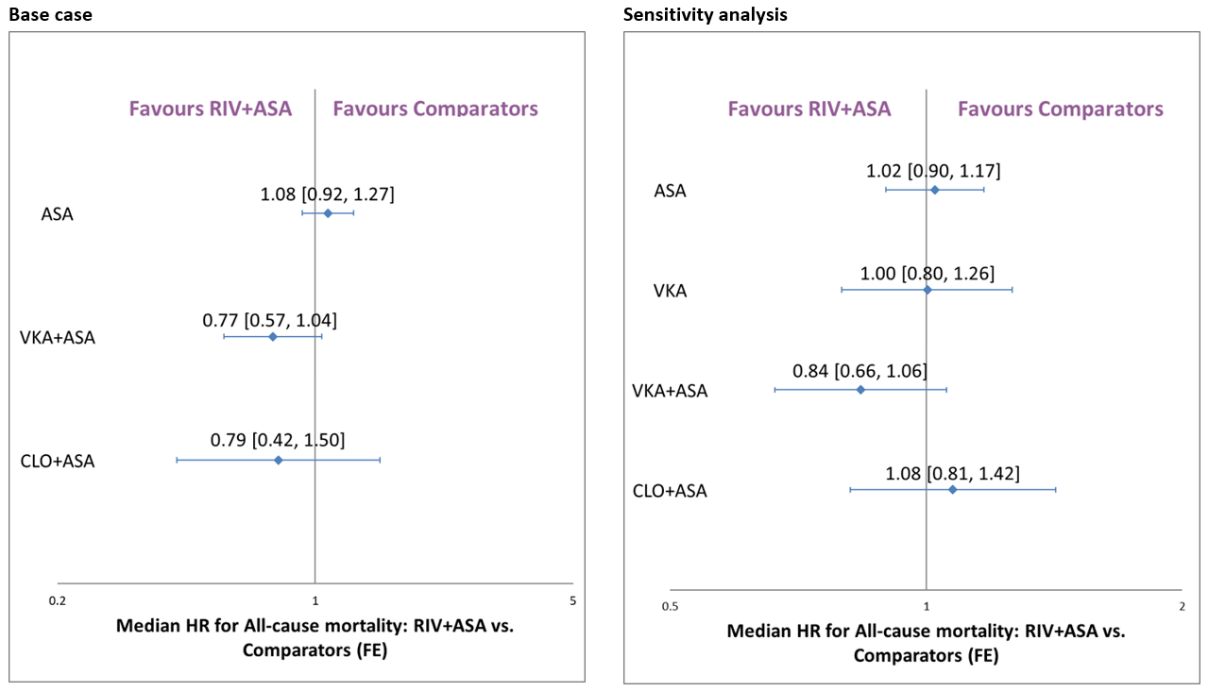


ASA, acetylsalicylic acid; FE, fixed effect; RIV, rivaroxaban.

Supplementary Table 6. Input data for the NMA of the risk of any stroke

| Study | Treatments | Event rate  n/N (%) | HR [95%CI] | Analysis | |
| --- | --- | --- | --- | --- | --- |
|  |  |  |  | **Base case** | **Sensitivity** |
| CAPRIE[7] | CLO | 81/3223 (2.5%) | 0.99 [0.73, 1.35] | ❌ | ✔ |
|  | ASA | 82/3229 (2.5%) | Reference |  |  |
| CASPAR[3] | CLO+ASA | NA/425 (NA%) | 1.02 [0.41, 2.57] | ✔ | ✔ |
|  | ASA | NA/426 (NA%) | Reference |  |  |
| Johnson, 2002&2004[11] | VKA+ASA | 31/418 (7.4%) | 1.06 [0.64, 1.76] | ✔ | ✔ |
|  | ASA | 29/413 (7.0%) | Reference |  |  |
| VOYAGER PAD[6] | RIV+ASA | 81/3286 (2.5%) | 0.94 [0.69, 1.27] | ✔ | ✔ |
|  | ASA | 86/3278 (2.6%) | Reference |  |  |
| BOA[5] | VKA | 35/1326 (2.6%) | 0.74 [0.48, 1.14] | ❌ | ✔ |
|  | ASA | 47/1324 (3.5%) | Reference |  |  |
| CHARISMA[4] | CLO+ASA | 36/1545 (2.3%) | 0.79 [0.51, 1.21] | ❌ | ✔ |
|  | ASA | 46/1551 (3.0%) | Reference |  |  |
| COMPASS[10] | RIV+ASA | 25/2492 (1.0%) | 0.54 [0.33, 0.87] | ❌ | ✔ |
|  | ASA | 47/2504 (1.9%) | Reference |  |  |
| WAVE[2] | VKA+ASA | 38/1080 (3.5%) | 1.01 [0.65, 1.59] | ❌ | ✔ |
|  | ASA | 38/1081 (3.5%) | Reference |  |  |

✔– study eligible for the analysis; ❌– study ineligible for the analysis

ASA, acetylsalicylic acid; CLO, clopidogrel; RIV, rivaroxaban; VKA, vitamin K antagonist.

Supplementary Figure 11. Networks of evidence for the risk of any stroke


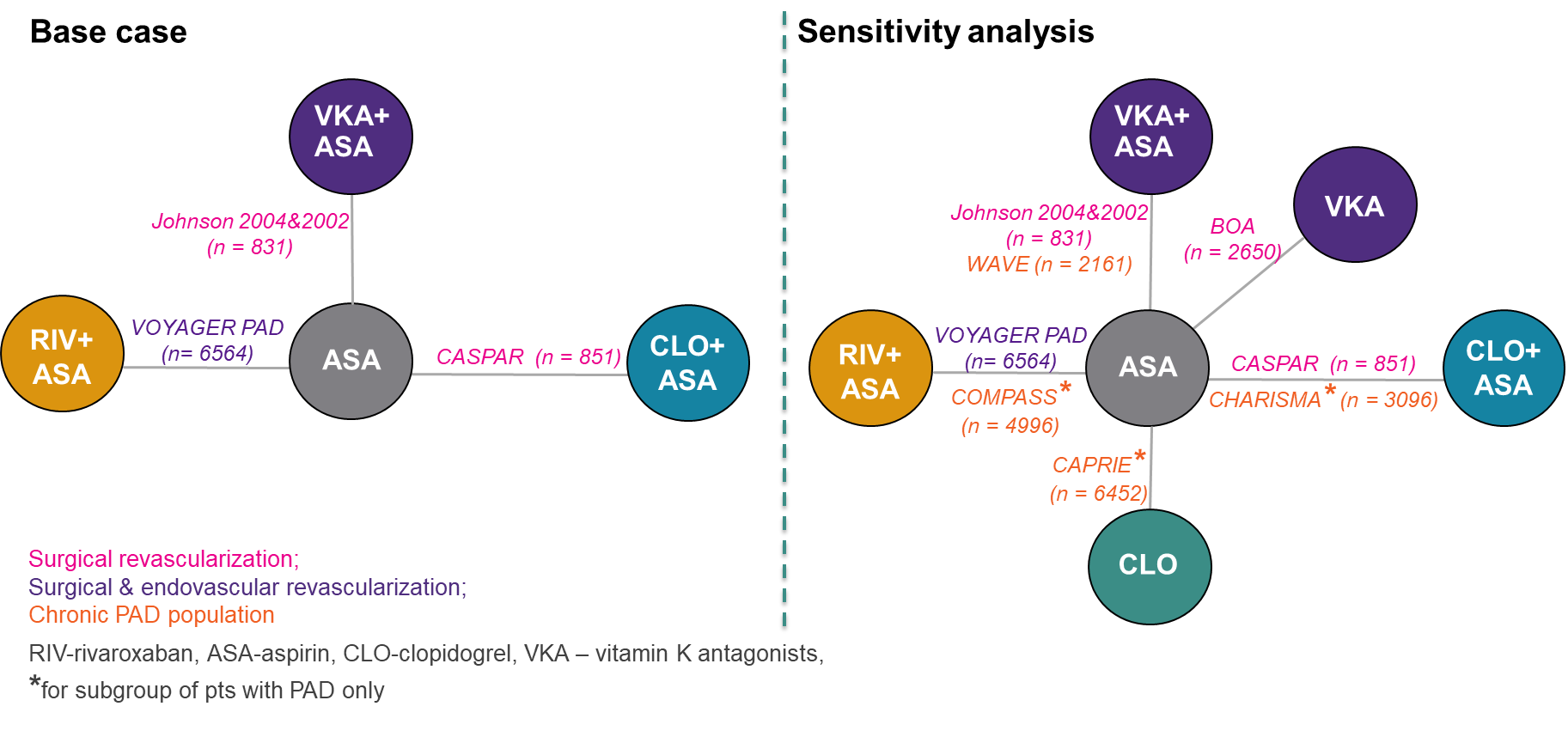


Supplementary Figure 12. Forest plots comparing RIV plus ASA versus comparators regarding any stroke


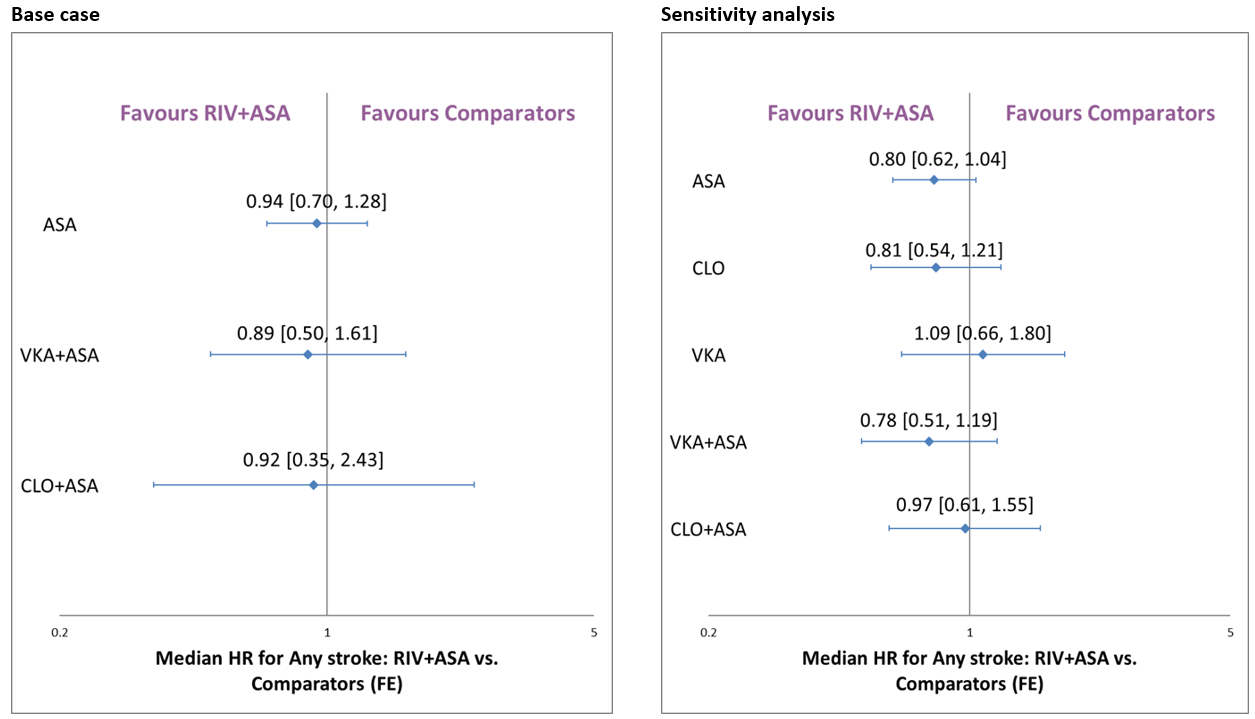


ASA, acetylsalicylic acid; FE, fixed effect; RIV, rivaroxaban.

Supplementary Table 7. Input data for the NMA of the risk of major bleeding

| Study | Treatments | Event rate  n/N (%) | HR [95%CI] | Analysis | | Definition |
| --- | --- | --- | --- | --- | --- | --- |
|  |  |  |  | **Base case** | **Sensitivity** |  |
| CASPAR[3] | CLO+ASA | 9/426 (2.1%) | 1.79 [0.60,5.35] | ✔ | ✔ | **GUSTO classification** of “severe bleeding” |
|  | ASA | 5/425 (1.2%) | Reference |  |  |  |
| Johnson, 2002&2004[11] | VKA+ASA | 35/418 (8.4%) | 2.36 [1.29,4.33] | ✔ | ✔ | Intracranial bleed, hospitalization for bleeding, an operation for control of bleeding, or a blood transfusion |
|  | ASA | 15/413 (3.6%) | Reference |  |  |  |
| VOYAGER PAD[6] | RIV+ASA | 62/3256 (1.9%) | 1.43 [0.97, 2.10] | ✔ | ✔ | TIMI |
|  | ASA | 44/3248 (1.4%) | Reference |  |  |  |
| BOA[5] | VKA | 108/1326 (8.1%) | 1.96 [1.42, 2.71] | ❌ | ✔ | Fatal  bleeding episodes, intracranial haemorrhage, or any bleeding  episode requiring hospital attendance, irrespective of  interventions |
|  | ASA | 56/1324 (4.2%) | Reference |  |  |  |
| CHARISMA[4] | CLO+ASA | 26/1545 (1.6%) | 0.97 [0.56, 1.66] | ❌ | ✔ | **GUSTO classification** of “severe bleeding” |
|  | ASA | 27/1551 (1.7%) | Reference |  |  |  |
| COMPASS[10] | RIV+ASA | 77/2492 (3.1%) | 1.61 [1.12, 2.31] | ❌ | ✔ | Modified **ISTH** major bleeding definition |
|  | ASA | 48/2504 (1.9%) | Reference |  |  |  |
| WAVE[2] | VKA+ASA | 43/1080 (4.0%) | 3.41 [1.84, 6.35] | ❌ | ✔ | Life-threatening bleeding |
|  | ASA | 13/1081 (1.2%) | Reference |  |  |  |

✔– study eligible for the analysis; ❌– study ineligible for the analysis

ASA, acetylsalicylic acid; CLO, clopidogrel; RIV, rivaroxaban; VKA, vitamin K antagonist.

Supplementary Figure 13. Networks of evidence for the risk of major bleeding


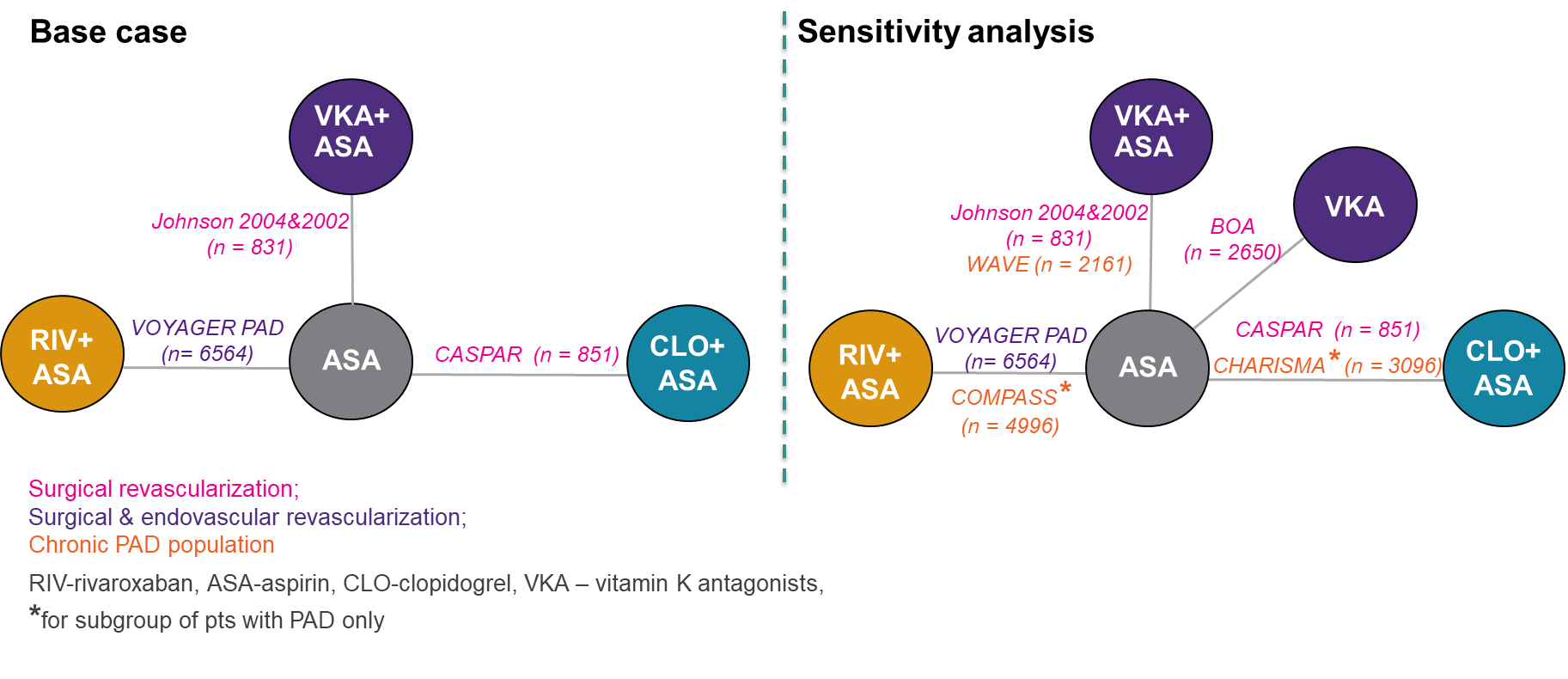


Supplementary Figure 14. Forest plots comparing RIV plus ASA versus comparators regarding major bleeding


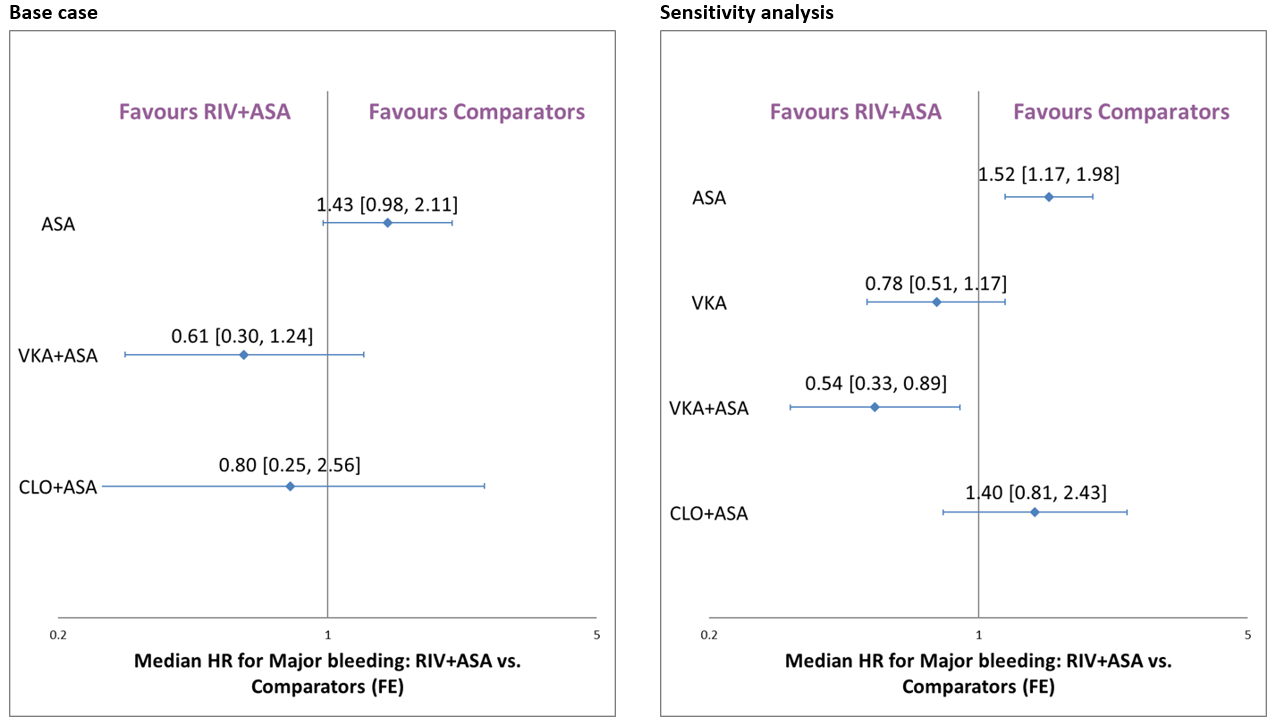


ASA, acetylsalicylic acid; FE, fixed effect; RIV, rivaroxaban.

Supplementary Table 8. Input data for the NMA of the risk of revascularisation

| Study | Treatments | Event rate  n/N (%) | HR [95%CI] | Analysis | |
| --- | --- | --- | --- | --- | --- |
|  |  |  |  | **Base case** | **Sensitivity** |
| MIRROR[14] | CLO+ASA | 2/40 (5.0%) | 0.23 [0.05, 1.08] | ✔ | ✔ |
|  | ASA | 8/40 (20.0%) | Reference |  |  |
| CASPAR[3] | CLO+ASA | NA/425 (NA%) | 0.89 [0.65, 1.23] | ✔ | ✔ |
|  | ASA | NA/426 (NA%) | Reference |  |  |
| VOYAGER PAD[6] | RIV+ASA | 584/3286 (17.8%) | 0.88 [0.79, 0.99] | ✔ | ✔ |
|  | ASA | 655/3278 (20.0%) | Reference |  |  |
| BOA[5] | VKA | 429/1326 (32.4%) | 0.95 [0.84, 1.09] | ❌ | ✔ |
|  | ASA | 446/1324 (33.7%) | Reference |  |  |
| CHARISMA[4] | CLO+ASA | 58/1545 (3.8%) | 0.73 [0.52, 1.03] | ❌ | ✔ |
|  | ASA | 79/1551 (5.1%) | Reference |  |  |
| COMPASS[10] | RIV+ASA | 242/2492 (9.7%) | 0.93 [0.78, 1.11] | ❌ | ✔ |
|  | ASA | 260/2504 (10.4%) | Reference |  |  |
| WAVE[2] | VKA+ASA | 36/1080 (3.3%) | 0.90 [0.57, 1.41] | ❌ | ✔ |
|  | ASA | 40/1081 (3.7%) | Reference |  |  |

✔– study eligible for the analysis; ❌– study ineligible for the analysis

ASA, acetylsalicylic acid; CLO, clopidogrel; RIV, rivaroxaban; VKA, vitamin K antagonist.

Supplementary Figure 15. Networks of evidence for the risk of revascularisation


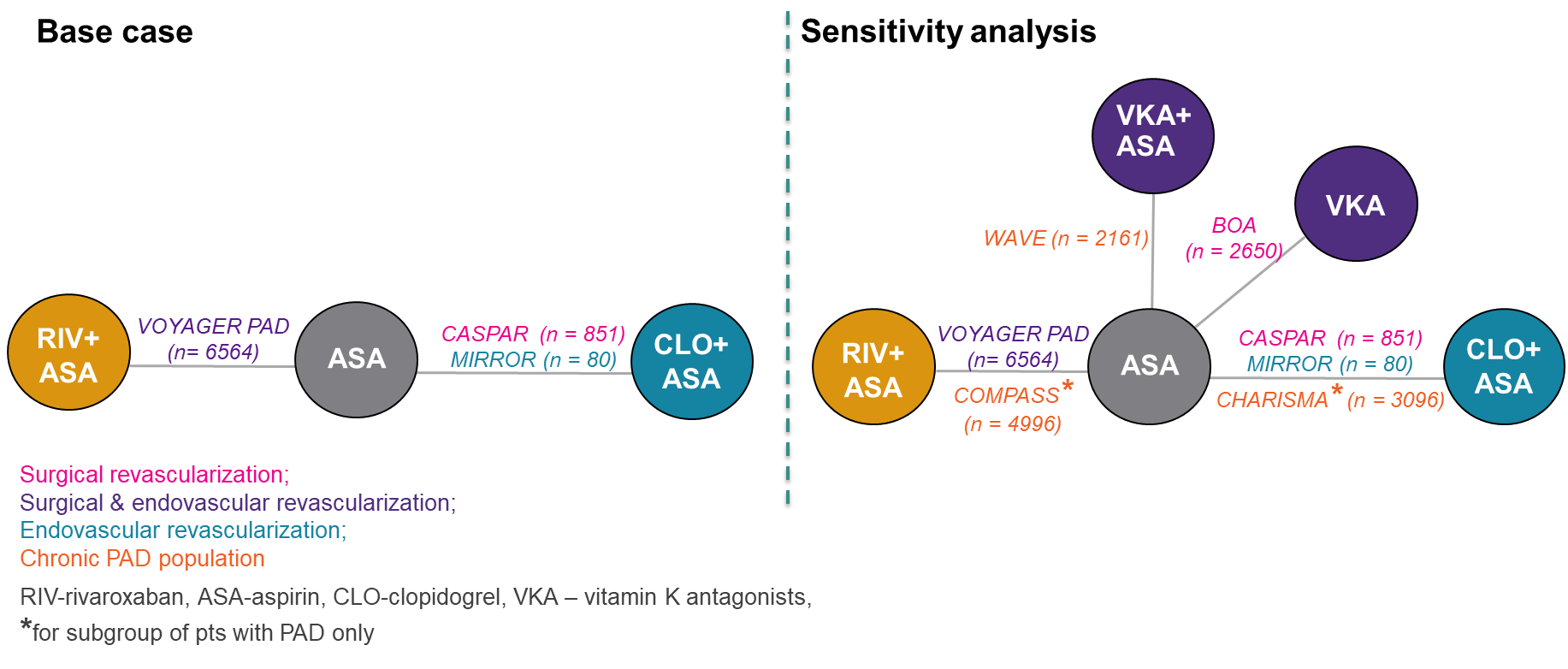


Supplementary Figure 16. Forest plots comparing RIV plus ASA versus comparators regarding revascularisation


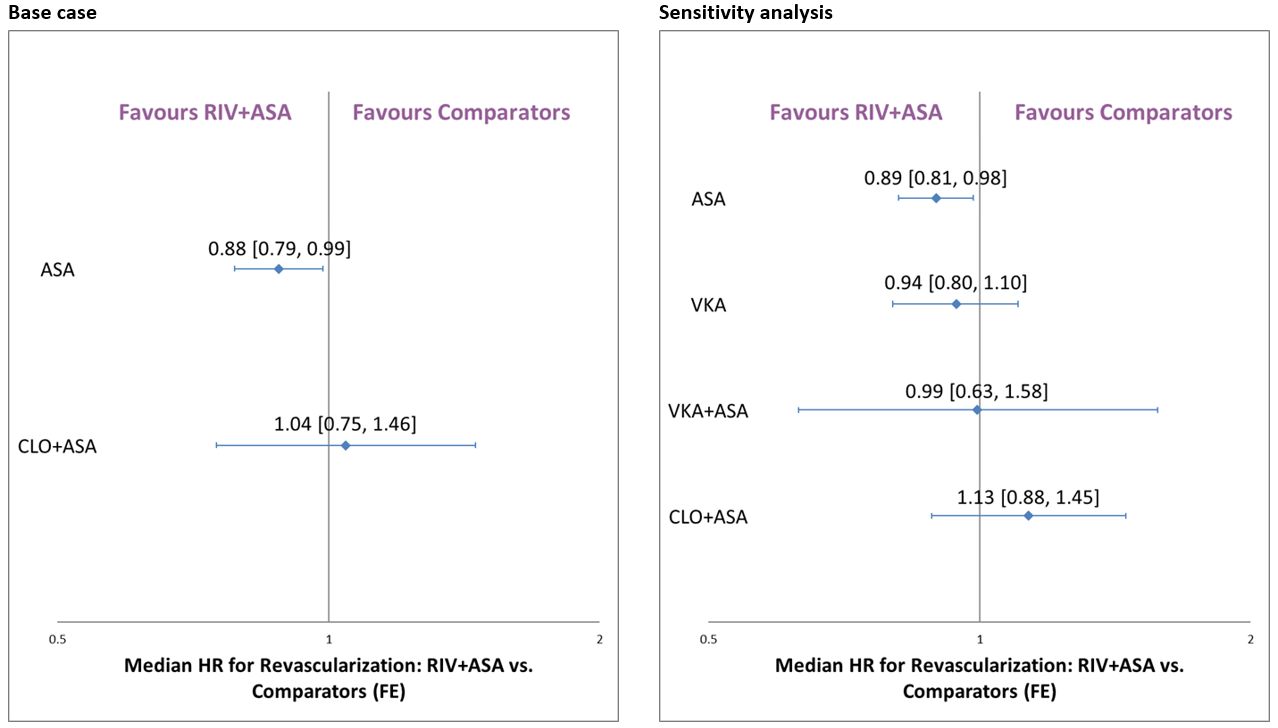


ASA, acetylsalicylic acid; FE, fixed effect; RIV, rivaroxaban.

**References to supplementary tables and figures**

1. Higgins JPT, Altman DG, Gøtzsche PC, et al. The Cochrane Collaboration’s tool for assessing risk of bias in randomised trials. BMJ. 2011;343:d5928.

2. Anand S, Yusuf S, Xie C, et al. Oral anticoagulant and antiplatelet therapy and peripheral arterial disease. N Engl J Med. 2007;357(3):217-27.

3. Belch JJ, Dormandy J, Biasi GM, et al. Results of the randomized, placebo-controlled clopidogrel and acetylsalicylic acid in bypass surgery for peripheral arterial disease (CASPAR) trial. J Vasc Surg. 2010;52(4):825-33, 833.e1-2.

4. Bhatt DL, Fox KA, Hacke W, et al. Clopidogrel and aspirin versus aspirin alone for the prevention of atherothrombotic events. N Engl J Med. 2006;354(16):1706-17.

5. Efficacy of oral anticoagulants compared with aspirin after infrainguinal bypass surgery (The Dutch Bypass Oral Anticoagulants or Aspirin Study): a randomised trial. Lancet. 2000;355(9201):346-51.

6. Bonaca MP, Bauersachs RM, Anand SS, et al. Rivaroxaban in Peripheral Artery Disease after Revascularization. N Engl J Med. 2020;382(21):1994-2004.

7. A randomised, blinded, trial of clopidogrel versus aspirin in patients at risk of ischaemic events (CAPRIE). CAPRIE Steering Committee. Lancet. 1996;348(9038):1329-39.

8. Cassar K, Ford I, Greaves M, et al. Randomized clinical trial of the antiplatelet effects of aspirin-clopidogrel combination versus aspirin alone after lower limb angioplasty. Br J Surg. 2005;92(2):159-65.

9. Eikelboom JW, Hankey GJ, Thom J, et al. Enhanced antiplatelet effect of clopidogrel in patients whose platelets are least inhibited by aspirin: a randomized crossover trial. J Thromb Haemost. 2005;3(12):2649-55.

10. Eikelboom JW, Connolly SJ, Bosch J, et al. Rivaroxaban with or without Aspirin in Stable Cardiovascular Disease. N Engl J Med. 2017;377(14):1319-1330.

11. Johnson WC, Williford WO. Benefits, morbidity, and mortality associated with long-term administration of oral anticoagulant therapy to patients with peripheral arterial bypass procedures: a prospective randomized study. J Vasc Surg. 2002;35(3):413-21.

12. Li H, Zhang F, Liang G, et al. A prospective randomized controlled clinical trial on clopidogrel combined with warfarin versus clopidogrel alone in the prevention of restenosis after endovascular treatment of the femoropopliteal artery. Ann Vasc Surg. 2013;27(5):627-33.

13. Liang GZ, Zhang FX, Luo XY, et al. [A prospective randomized control clinical trial about clopidogrel combined with warfarin versus clopidogrel alone in the prevention of restenosis after femoral-popliteal artery angioplasty]. Zhonghua Wai Ke Za Zhi. 2012;50(8):704-8. In Chinese.

14. Tepe G, Bantleon R, Brechtel K, et al. Management of peripheral arterial interventions with mono or dual antiplatelet therapy--the MIRROR study: a randomised and double-blinded clinical trial. Eur Radiol. 2012;22(9):1998-2006.
