# Supplementary material for: Efficacy and Safety of Rivaroxaban Compared with Other Therapies Used in Patients with Peripheral Artery Disease Undergoing Peripheral Revascularization: A Systematic Literature Review and Network Meta-Analysis
Source: Cardiovasc Ther. 2021 Aug 24;2021:8561350. doi: 10.1155/2021/8561350 (PMC8407972; doi:10.1155/2021/8561350)
Supplement: Supplementary Materials — Supplementary Table 1: credibility assessment of trials included in the NMA [1]. Supplementary Table 2: input data for the NMA of the risk of myocardial infarction. Supplementary Table 3: input data for the NMA of the risk of ischemic stroke. Supplementary Table 4: input data for the NMA of the risk of cardiovascular death. Supplementary Table 5: input data for the NMA of the risk of all-cause mortality. Supplementary Table 6: input data for the NMA of the risk of any stroke. Supplementary Table 7: input data for the NMA of the risk of major bleeding. Supplementary Table 8: input data for the NMA of the risk of revascularization. Supplementary Figure 1: networks of evidence for the risk of amputations. Supplementary Figure 2: forest plots comparing RIV plus ASA versus comparators regarding amputation. Supplementary Figure 3: networks of evidence for the risk of myocardial infarction. Supplementary Figure 4: forest plots comparing RIV plus ASA versus comparators regarding myocardial infarction. Supplementary Figure 5: networks of evidence for the risk of ischemic stroke. Supplementary Figure 6: forest plots comparing RIV plus ASA versus comparators regarding ischemic stroke. Supplementary Figure 7: networks of evidence for the risk of cardiovascular death. Supplementary Figure 8: forest plots comparing RIV plus ASA versus comparators regarding cardiovascular death. Supplementary Figure 9: networks of evidence for the risk of all-cause mortality. Supplementary Figure 10: forest plots comparing RIV plus ASA versus comparators regarding all-cause mortality. Supplementary Figure 11: networks of evidence for the risk of any stroke. Supplementary Figure 12: forest plots comparing RIV plus ASA versus comparators regarding any stroke. Supplementary Figure 13: networks of evidence for the risk of major bleeding. Supplementary Figure 14: forest plots comparing RIV plus ASA versus comparators regarding major bleeding. Supplementary Figure 15: networks of evidence for the risk o [file 8561350.f1.zip › Supplementary Deascription.docx]

Supplementary Deascription:

Supplementary Table 1. Credibility assessment of trials included in the NMA[1]

Supplementary Table 2. Input data for the NMA of the risk of myocardial infarction

Supplementary Table 3. Input data for the NMA of the risk of ischaemic stroke

Supplementary Table 4. Input data for the NMA of the risk of cardiovascular death

Supplementary Table 5. Input data for the NMA of the risk of all-cause mortality

Supplementary Table 6. Input data for the NMA of the risk of any stroke

**Supplementary Table 7. Input data for the NMA of the risk of major bleeding**

**Supplementary Table 8. Input data for the NMA of the risk of revascularization**

Supplementary Figure 1. Networks of evidence for the risk of amputations

Supplementary Figure 2. Forest plots comparing RIV plus ASA versus comparators regarding amputation

Supplementary Figure 3. Networks of evidence for the risk of myocardial infarction

Supplementary Figure 4. Forest plots comparing RIV plus ASA versus comparators regarding myocardial infarction

Supplementary Figure 5. Networks of evidence for the risk of ischaemic stroke

Supplementary Figure 6. Forest plots comparing RIV plus ASA versus comparators regarding ischaemic stroke

Supplementary Figure 7. Networks of evidence for the risk of cardiovascular death

Supplementary Figure 8. Forest plots comparing RIV plus ASA versus comparators regarding cardiovascular death

Supplementary Figure 9. Networks of evidence for the risk of all-cause mortality

Supplementary Figure 10. Forest plots comparing RIV plus ASA versus comparators regarding all-cause mortality

Supplementary Figure 11. Networks of evidence for the risk of any stroke

Supplementary Figure 12. Forest plots comparing RIV plus ASA versus comparators regarding any stroke

Supplementary Figure 13. Networks of evidence for the risk of major bleeding

Supplementary Figure 14. Forest plots comparing RIV plus ASA versus comparators regarding major bleeding

Supplementary Figure 15. Networks of evidence for the risk of revascularisation

Supplementary Figure 16. Forest plots comparing RIV plus ASA versus comparators regarding revascularisation
